# Design, synthesis and biological evaluation of novel thiazole-naphthalene derivatives as potential anticancer agents and tubulin polymerisation inhibitors

**DOI:** 10.1080/14756366.2021.1958213

**Published:** 2021-07-26

**Authors:** Guangcheng Wang, Wenjing Liu, Meiyan Fan, Min He, Yongjun Li, Zhiyun Peng

**Affiliations:** a State Key Laboratory of Functions and Applications of Medicinal Plants, Guizhou Provincial Key Laboratory of Pharmaceutics, Guizhou Medical University, Guiyang, China; b School of Pharmacy, Guizhou Medical University, Guiyang, China; c Engineering Research Center for the Development and Application of Ethnic Medicine and TCM (Ministry of Education), Guizhou Medical University, Guiyang, China; d College of Food Science and Technology, Shanghai Ocean University, Shanghai, China

**Keywords:** Thiazole, naphthalene, tubulin, anticancer, tubulin polymerisation

## Abstract

A novel series of thiazole-naphthalene derivatives as tubulin polymerisation inhibitors were designed, synthesised, and evaluated for the anti-proliferative activities. The majority of the tested compounds exhibited moderate to potent antiproliferative activity on the MCF-7 and A549 cancer cell lines. Among them, compound **5b** was found to be the most active compound with IC_50_ values of 0.48 ± 0.03 and 0.97 ± 0.13 μM. Moreover, mechanistic studies revealed that **5b** significantly inhibited tubulin polymerisation with an IC_50_ value of 3.3 µM, as compared to the standard drug colchicine (IC_50_ = 9.1 μM). Further cellular mechanism studies elucidated that **5b** arrested the cell cycle at G2/M phase and induced apoptosis in MCF-7 cancer cells. Molecular modelling study indicated that **5b** binds well to the colchicine binding site of tubulin. In summary, these results suggest that **5b** represents a promising tubulin polymerisation inhibitor worthy of further investigation as potential anticancer agents.

## 1. Introduction

Microtubules are a component of the cytoskeleton, which is formed through the polymerisation of α- and β-tubulin heterodimers^1^. They play an important role in a wide range of cellular processes such as cell proliferation, cellular transport, intracellular trafficking, and angiogenesis^2–4^. In the cellular microtubule system, microtubule formation is a dynamic equilibrium related to the polymerisation and depolymerisation of α, β-tubulin heterodimers^5^. Disrupting the tubulin dynamics equilibrium blocks the cell division at mitosis and thus resulting in the cell cycle arrest at metaphase, which leads to cell death^6^^,^^7^. Moreover, cancer cells are more in the division phase than normal cells, which means that they are more prone to anti-tubulin agents^8^. Therefore, microtubules have become an attractive target for the design and development of novel anticancer agents^9–11^.

**Figure 1.**
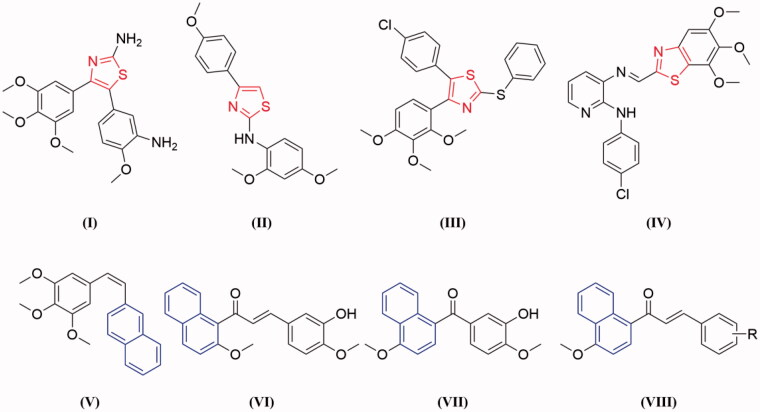
Some tubulin polymerisation inhibitors containing thiazole or naphthalene moiety.

Thiazole is an important heterocyclic scaffold widely found in a range of synthetic bioactive molecules, which has attracted considerable attention in drug discovery over the past decade. Thiazole derivatives displayed a wide range of pharmacological activities, such as anticancer, anti-inflammatory, antioxidant, antimicrobial, anti-HIV, and antibacterial activities^12–14^. Several thiazole-containing drugs have been approved for clinical use, such as sulphathiazole, ravuconazole, ritonavir, and meloxicam. It's important to note that thiazole could be used as a promising scaffold for the development of anticancer agents^15–17^. Over the last few years, numerous thiazole derivatives have been reported to show potent anticancer activity by inhibiting tubulin polymerisation (Figure 1, I–IV)^18–21^.

On the other hand, naphthalene is a prominent core structure in many anticancer agents. A number of naphthalene derivatives have been reported as potent inhibitors of tubulin (Figure 1, V–VIII)^22–25^. Such as Maya et al. reported the synthesis of a series of new naphthalene analogues of combretastatin A-4 (CA-4) and the most cytotoxic naphthalene analogues **V** exerted tubulin polymerisation inhibition activity and arrest cell cycle in G2/M phase in human cancer cells^25^. Based on the lead compound HMNC-74, we synthesised a series of new naphthalene-chalcone derivatives and evaluated their anticancer activity. Among them, compound **IV** was the most potent tubulin polymerisation inhibitor with an IC_50_ value of 8.4 μM^23^. Recently, we have also reported a new series of benzophenone derivatives bearing naphthalene moiety, and compound **VII** displayed potent antiproliferative activity against various cancer cell lines by targeting tubulin colchicine binding site^24^. Furthermore, we also designed a new series of chalcones containing naphthalene moiety (**VIII**) based on natural tubulin inhibitor millepachine^22^^,^^26^.

**Figure 2.**
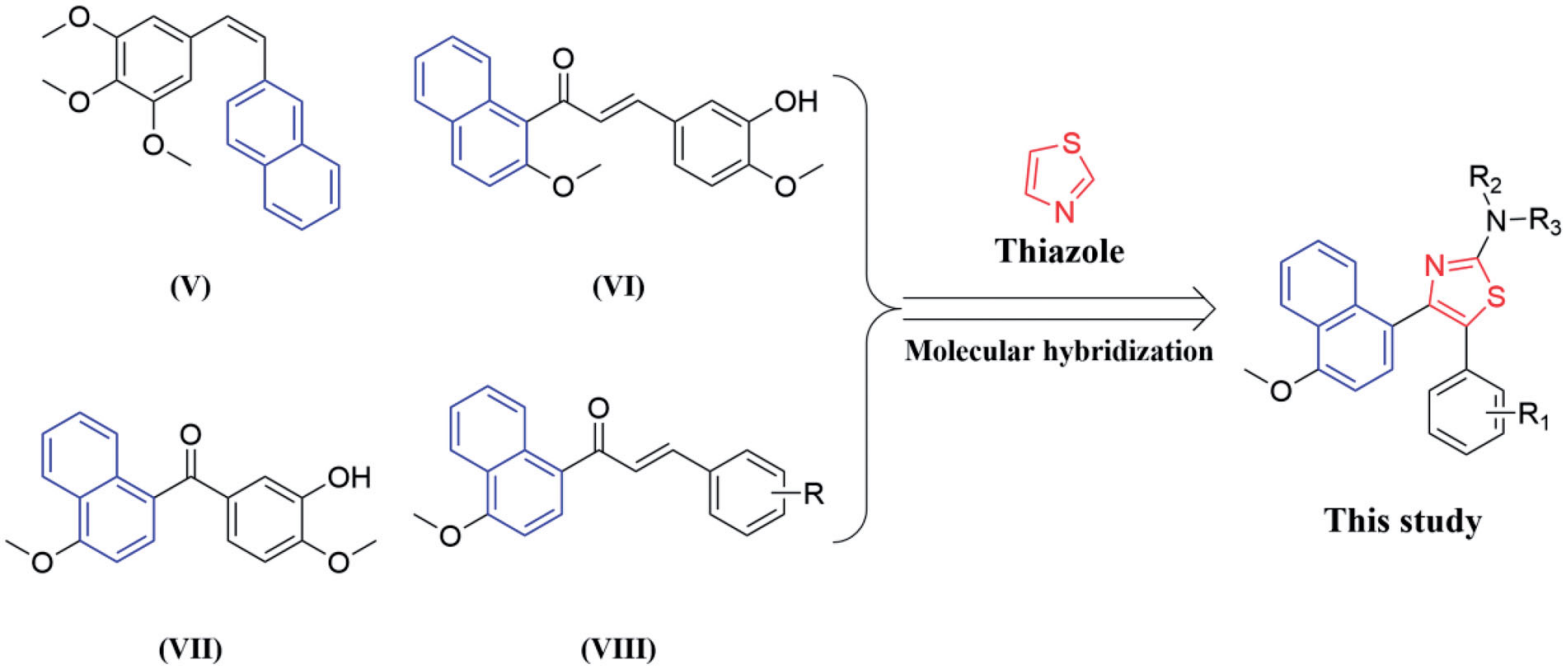
The design strategy of the target compounds in this study.

Molecular hybridisation approach is an efficient and often used method for the design and development of new therapeutic agents in the current medicinal chemistry research^27–30^. Encouraged by these observations and in continuation of our interest in the discovery of tubulin inhibitors, herein we reported the synthesis of a novel series of thiazole-naphthalene derivatives by hybridisation of thiazole moiety with naphthalene structural motif into a single molecule and examined their antiproliferative and antitubulin effects (Figure 2).

## 2. Chemistry

**Scheme 1.**
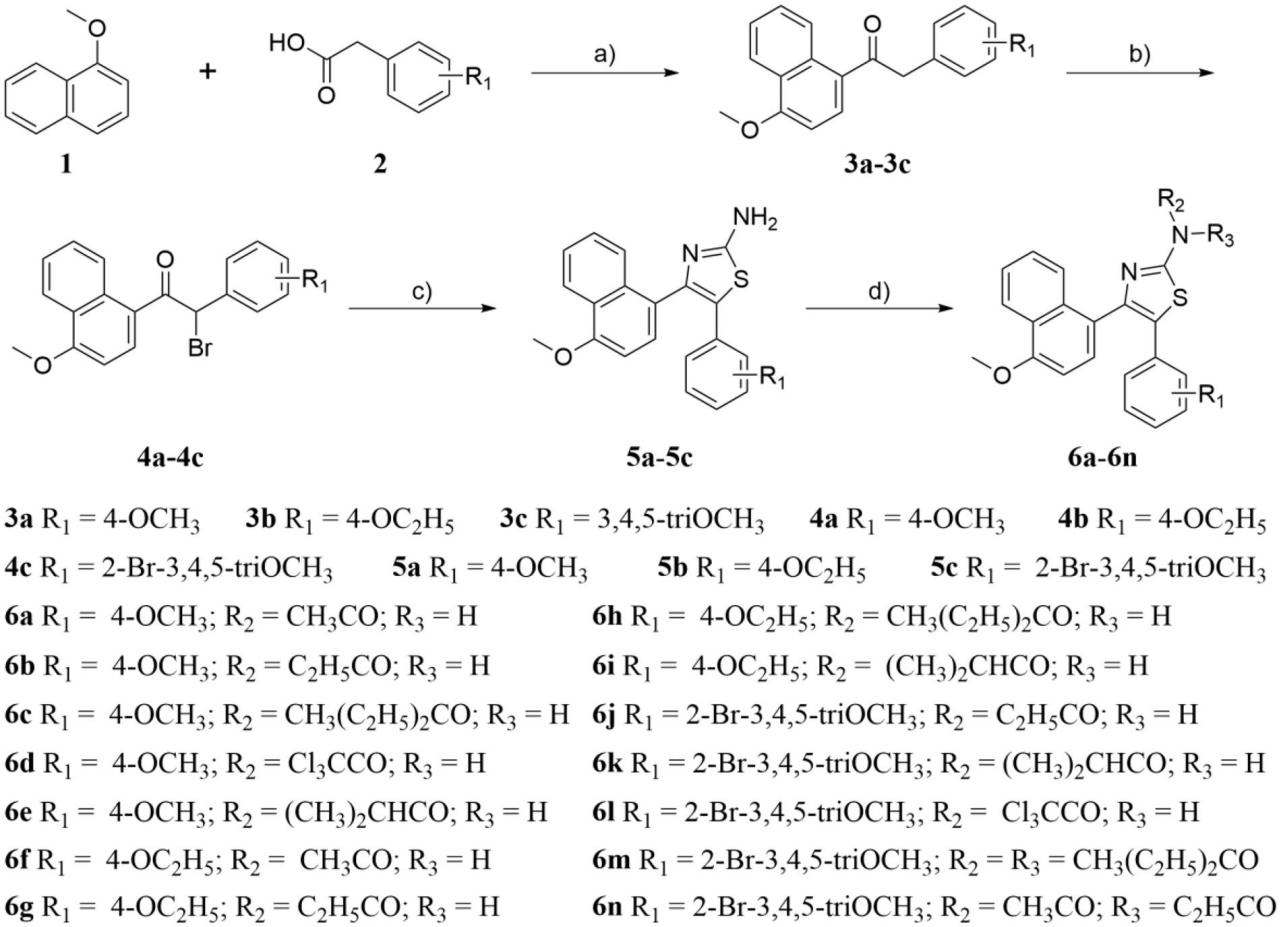
Reagents and conditions: (a) TFAA, TFA, room temperature, 12 h; (b) Pyridinium tribromide, CH_2_Cl_2_, r.t. 1 h; (c) Thiourea, EtOH, reflux, 3 h; (d) Acid anhydride, 120 °C, 3 h.

A series of thiazole-naphthalene derivatives (**5a–5c** and **6a–6n**) were synthesised according to the pathways described in Scheme 1. Deoxybenzoins **3a–3c** was prepared by condensation of 1-methoxynaphthalene **1** with appropriate phenylacetic acids **2** in the presence of trifluoroacetic anhydride (TFAA) in trifluoroacetic acid (TFA) at room temperature. Treatment of **3a–3c** with pyridinium tribromide in CH_2_Cl_2_ to give compounds **4a–4c**. Then, condensation of **4a–4c** with thiourea under reflux in ethanol to afford the title compounds (**5a–5c**) in high yields. Finally, a series of thiazole-naphthalene derivatives (**6a–6n**) were prepared by a condensation reaction of **5a–5c** with a variety of commercially available acid anhydride. All of the title compounds are new and not reported in the literature to date.

The structures of all the title compounds (**5a–5c** and **6a–6n**) were characterised by ^1^H NMR, ^13 ^C NMR, and HRMS (Supplementary material). For instance, the ^1^H NMR spectrum of compound **5a** shown two singlets at *δ* 3.68 and *δ* 3.99 due to two methoxy groups in the aromatic ring. The single peak of NH_2_ group was observed at *δ* 5.59 ppm. Two doublet peaks at *δ* 6.59 and *δ* 6.94 with coupling constant of 8.8 Hz were attributed to the aromatic protons of C3,5-H and C2,6-H of the 4-methoxyphenyl moiety, respectively. The six protons of 4-methoxynaphthalen-1-yl moiety appeared as multiplet or doublet peak (*J* = 8.0 Hz) in the region of *δ* 6.72–6.74 ppm and *δ* 7.42–8.29 ppm. Besides, the ^13 ^C NMR spectrum of compound **5a** shown three peaks at *δ* 157.38–167.76 ppm, which attributed to the aromatic carbon connected with the amino or methoxy group. The signals observed at *δ* 103.78–146.16 ppm were assigned to aromatic carbons in the compound. The peaks of two methoxy groups were observed at *δ* 55.30 ppm and *δ* 55.84 ppm, respectively. Furthermore, HRMS of compound **5a** showed a molecular ion peak at m/z 363.1126 as [M + H]^+^ which also supports the proposed structure of the compound.

## 3. Biological evaluation

### 3.1. Antiproliferative activity

All newly prepared compounds were tested for their antiproliferative activity on the human breast cancer cell line (MCF-7) and human lung adenocarcinoma cell line (A549) using the CCK-8 assay with cisplatin, 5-fluorouracil, tamoxifen, and CA-4 as the reference drugs. The results are expressed as IC_50_ values (50% inhibitory concentrations) and are summarised in Table 1. Except for compounds **5c**, **6j**, **6k,** and **6 m**, the majority of the tested compounds exhibited moderate to potent antiproliferative activity. Among these compounds, compounds **5a**, **5b**, **6a**, **6d**, and **6l** displayed potent antiproliferative activity as compared to the standard drugs (cisplatin, 5-fluorouracil, tamoxifen, and CA-4). All other tested compounds (**6b**, **6c**, **6e–6j**, and **6n**) shown moderate inhibitory activity.

**Table 1.** Antiproliferative activities of target compounds (**5a–5c** and **6a–6n**) on the human cancer cell lines.

Compound	R	IC_50_ (μM)^a^	
		MCF-7	A549
**5a**	R_1_ = 4-OCH_3_	1.80 ± 0.07	2.72 ± 0.18
**5b**	R_1_ = 4-OC_2_H_5_	0.48 ± 0.03	0.97 ± 0.13
**5c**	R_1_ = 2-Br-3,4,5-triOCH_3_	31.05 ± 0.70	>50.0
**6a**	R_1_ = 4-OCH_3_; R_2_ = CH_3_CO; R_3_ = H	2.16 ± 0.27	16.14 ± 0.83
**6b**	R_1_ = 4-OCH_3_; R_2_ = C_2_H_5_CO; R_3_ = H	14.75 ± 0.45	40.32 ± 0.32
**6c**	R_1_ = 4-OCH_3_; R_2_ = CH_3_(C_2_H_5_)_2_CO; R_3_ = H	14.90 ± 0.34	>50.0
**6d**	R_1_ = 4-OCH_3_; R_2_ = Cl_3_CCO; R_3_ = H	1.03 ± 0.09	1.91 ± 0.08
**6e**	R_1_ = 4-OCH_3_; R_2_ = (CH_3_)_2_CHCO; R_3_ = H	12.27 ± 0.54	>50.0
**6f**	R_1_ = 4-OC_2_H_5_; R_2_ = CH_3_CO; R_3_ = H	5.49 ± 0.84	7.00 ± 0.30
**6g**	R_1_ = 4-OC_2_H_5_; R_2_ = C_2_H_5_CO; R_3_ = H	9.61 ± 0.54	18.67 ± 0.89
**6h**	R_1_ = 4-OC_2_H_5_; R_2_ = CH_3_(C_2_H_5_)_2_CO; R_3_ = H	7.18 ± 0.78	>50.0
**6i**	R_1_ = 4-OC_2_H_5_; R_2_ = (CH_3_)_2_CHCO; R_3_ = H	7.69 ± 0.34	>50.0
**6j**	R_1_ = 2-Br-3,4,5-triOCH_3_; R_2_ = C_2_H_5_CO; R_3_ = H	>50.0	>50.0
**6k**	R_1_ = 2-Br-3,4,5-triOCH_3_; R_2_ = (CH_3_)_2_CHCO; R_3_ = H	>50.0	>50.0
**6l**	R_1_ = 2-Br-3,4,5-triOCH_3_; R_2_ = Cl_3_CCO; R_3_ = H	0.61 ± 0.06	0.92 ± 0.02
**6m**	R_1_ = 2-Br-3,4,5-triOCH_3_; R_2_ = R_3_ = CH_3_(C_2_H_5_)_2_CO	>50.0	>50.0
**6n**	R_1_ = 2-Br-3,4,5-triOCH_3_; R_2_ = CH_3_CO; R_3_= C_2_H_5_CO	13.49 ± 0.82	16.31 ± 0.89
**Cisplatin**	–	11.15 ± 0.75	4.92 ± 0.56
**5-Fu**	–	11.61 ± 0.60	2.75 ± 0.31
**Tamoxifen**	–	14.28 ± 0.40	20.20 ± 0.65
**CA-4**	–	5.55 ± 0.11	0.029 ± 0.004

^a^The values given are means of three experiments.

Based on the antiproliferative activities of these compounds, the structure-activity relationship (SAR) of this class of compounds is summarised. Compared the antiproliferative activity of **5a**, **5b,** and **5c**, the result was shown that the change of substituent affects the inhibitory activity. When the ethoxy group located at the para position of phenyl ring, the compound has the strongest activity (**5b**). The replacement of ethoxy group (**5b**) with methoxy group (**5a**) decreased the activity slightly. However, the replacement of 4-ethoxyphenyl (**5b**) with 2-bromo-3,4,5-trimethoxyphenyl (**5c**) resulted in a remarkable decrease of antiproliferative activity. Among this series of thiazole-naphthalene derivatives, compound **5b** with an ethoxy group at the 4-position of the phenyl ring and free amine group at the thiazole ring, was found to be the most active compound. On the other hand, introduction of fatty acyl group into the aminothiazole resulting in significantly decreased antiproliferative activity, such as CH_3_CO, C_2_H_5_CO, CH_3_(C_2_H_5_)_2_CO, and (CH_3_)_2_CHCO. It is interesting to point out that **6d** and **6l** containing the trichloroacetyl group (Cl_3_CCO) at the aminothiazole moiety significantly improved the antiproliferative activity. In summary, the information of SAR provided us a guideline to improve the antiproliferative activity in future structural modification.

In order to evaluate the safety of this series of compounds, the most potent compound **5b** was selected to tested its inhibitory activity against normal human embryonic kidney cell line (HEK293). The results showed that compound **5b** showed low toxicity in human normal cell line (IC_50_ = 16.37 ± 4.61 μM) compared to MCF-7 (IC_50_ = 0.48 ± 0.03 μM) and A549 (IC_50_ = 0.97 ± 0.13 μM) cancer cell lines. Hence, these compounds have good safety for potential application in the treatment of cancer.

### 3.2. *In vitro* tubulin polymerisation assay

**Figure 3.**
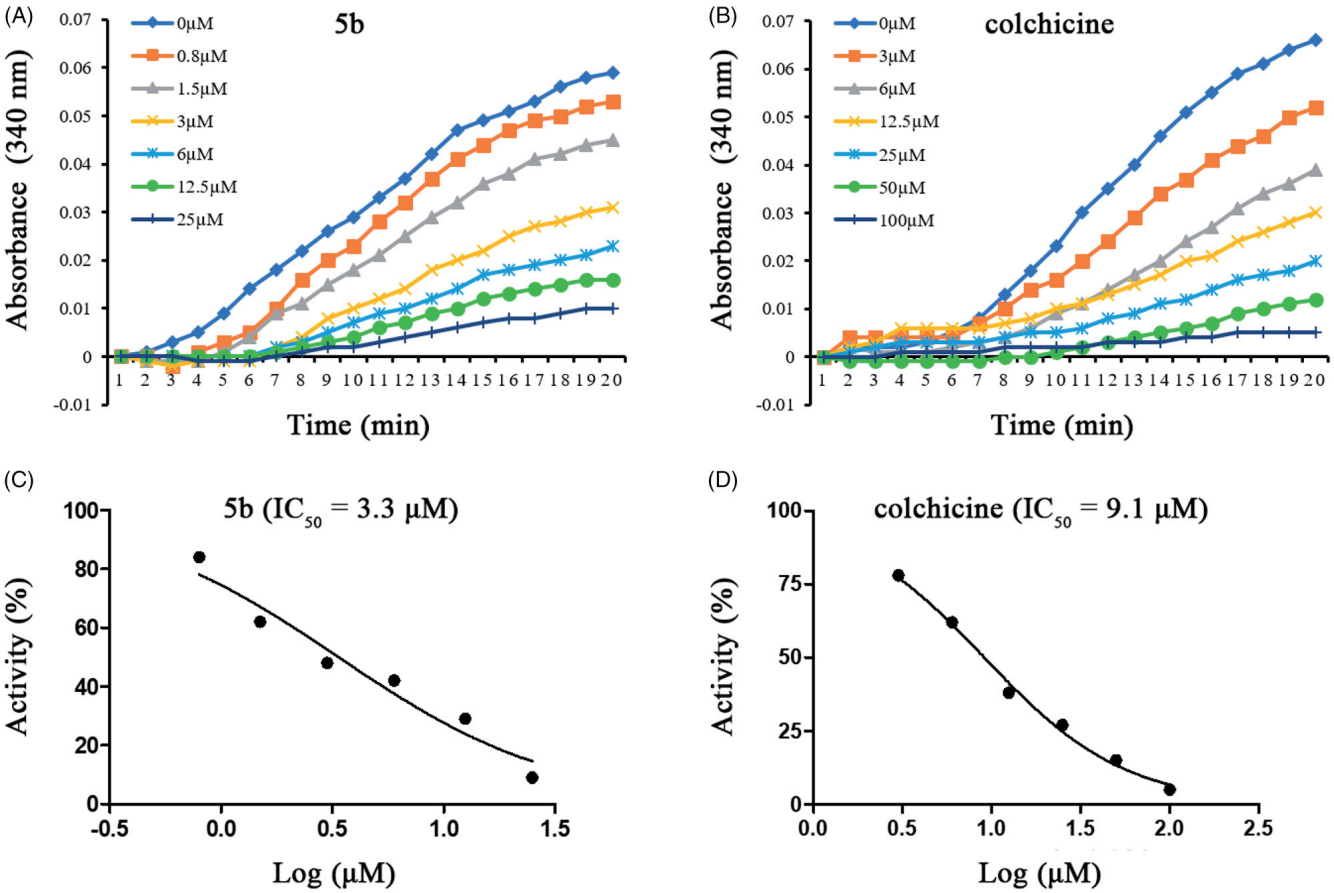
Tubulin polymerisation inhibitory activities of **5b** and colchicine. Purified tubulin protein was incubated at 37 °C in the absence or presence of **5b** (A,C) and colchicine (B,D) at the indicated concentrations.

To evaluate whether this series of compounds displayed anticancer activity by targeting tubulin-microtubule system, the *in vitro* tubulin polymerisation inhibitory activities of compounds **5b**, **6d**, and **6l** were evaluated using the method previously described^24^. Meanwhile, colchicine (tubulin destabilising agent) was chosen as the positive control. As shown in Table 2, the tubulin inhibitory polymerisation (IC_50_) of compounds **5b**, **6d**, and **6l** were 3.3 μM, 6.6 μM, and 4.0 μM, respectively. Considering the results of antiproliferative and *in vitro* tubulin inhibition activities, compound **5b** was selected to further discuss the mechanism of tubulin inhibition. As shown in Figure 3, compound **5b** exhibited similar action to that of colchicine. With the increase of the concentration of compound **5b** or colchicine, the fluorescence intensity of tubulin was obviously slowed down as compared with the control. These results indicated that **5b** was the tubulin destabilising agent with an IC_50_ value of 3.3 μM, which was superior to that of colchicine (IC_50_ = 9.1 μM). These data confirmed that the antiproliferative activity of these compounds is related to their inhibition of tubulin polymerisation, and tubulin was the most likely the target of these compounds.

**Table 2.** Tubulin inhibitory activities of compounds **5b**, **6d**, and **6l**.

Compound	Structure	IC_50_ (μM)
**5b**	R_1_ = 4-OC_2_H_5_	3.3
**6d**	R_1_ = 4-OCH_3_; R_2_ = Cl_3_CCO; R_3_ = H	6.6
**6l**	R_1_ = 2-Br-3,4,5-triOCH_3_; R_2_ = Cl_3_CCO; R_3_ = H	4.0
**Colchicine**		9.1

### 3.3. Cell cycle analysis

**Figure 4.**
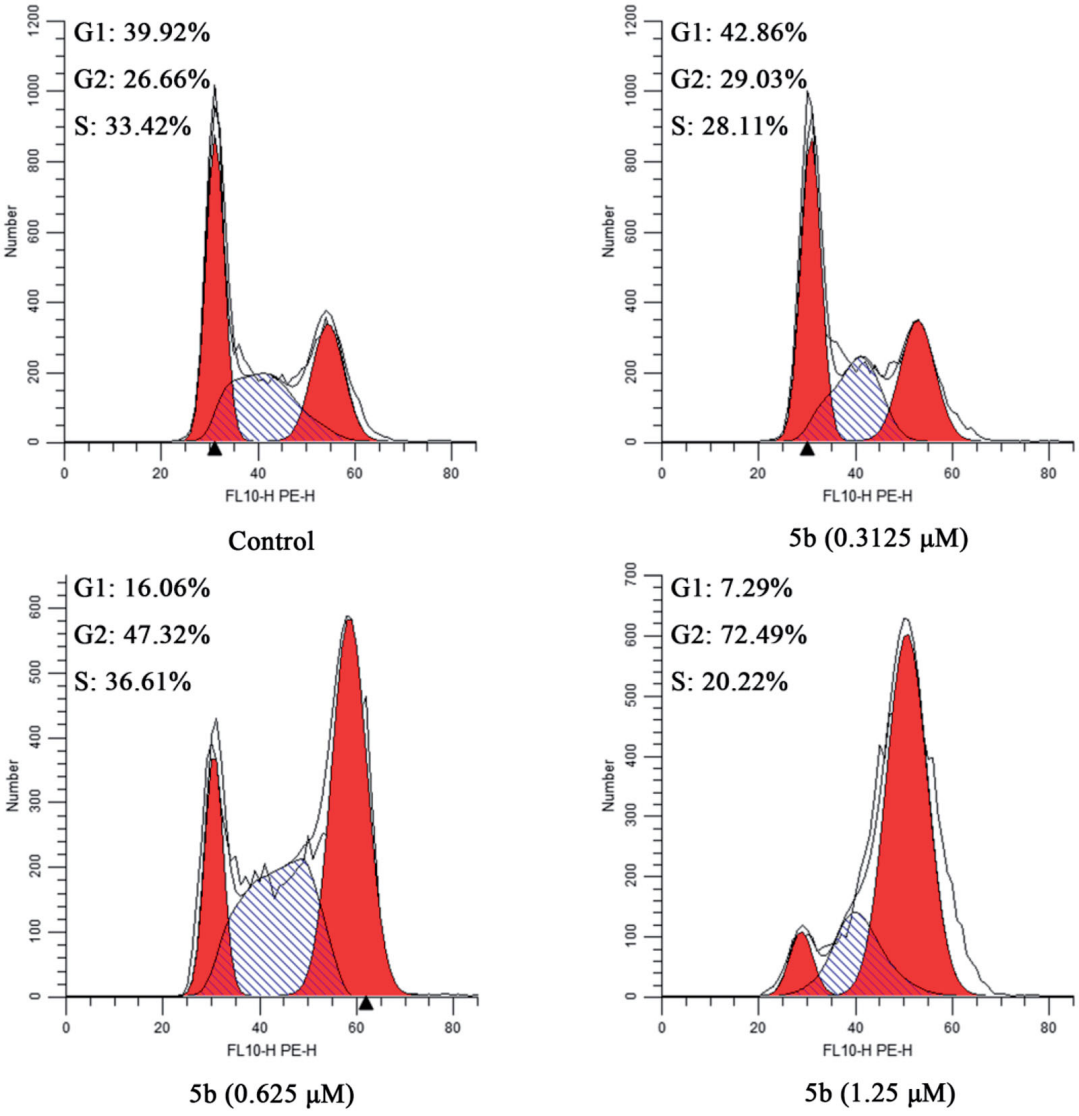
The cell cycle of MCF-7 cancer cells treated with different concentration of **5b** (0, 0.3125, 0.625, and 1.25 μM).

Based on the literature report, most tubulin destabilising agents can disrupt regulated cell cycle distribution and lead to cell cycle arrest at metaphase^23^^,^^31^. Therefore, we evaluated the effect of the most promising compound **5b** on the cell cycle of MCF-7 cancer cells using the flow cytometry analysis assay. In this study, MCF-7 cancer cells were incubated with different concentrations of **5b** (0.3125, 0.625, and 1.25 μM) for 24 h. The results were shown in Figure 4, after the addition of different concentrations of **5b** (0.3125, 0.625, and 1.25 μM), the G2/M population increased from 26.66% (control) to 72.49% (1.25 μM). These findings demonstrated that **5b** can arrest cell cycle at G2/M phase in a dose-dependent manner.

### 3.4. Induction of cellular apoptosis

**Figure 5.**
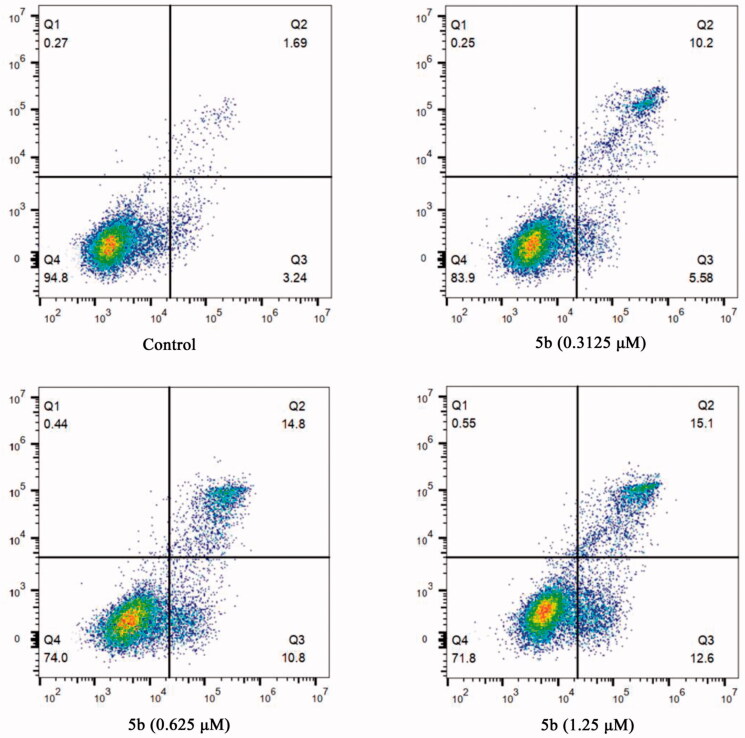
Flow cytometric analysis of apoptotic cells after treatment of MCF-7 cells with different concentrations of **5b** (0, 0.3125, 0.625, and 1.25 μM).

Considering that tubulin polymerisation inhibitors can induce cellular apoptosis^32^, the Annexin V-FITC/PI assay was performed to detect whether compound **5b** was able to induce cancer cell apoptosis. After treatment with different concentrations of **5b** (0, 0.3125, 0.625, and 1.25 μM), the obtained data showed **5b** can initiate cellular apoptosis and dissipate cellular integrity. As shown in Figure 5, when MCF-7 cancer cells were incubated with **5b** at 0.3125, 0.625, or 1.25 μM for 24 h, the total numbers of early and late apoptotic cells were 15.78%, 25.6%, and 27.7%, respectively, whereas that of the control group was only 4.93%. These results implied that compound **5b** effectively induced cell apoptosis in MCF-7 cells via a dose-dependent manner.

### 3.5. Molecular docking

**Figure 6.**
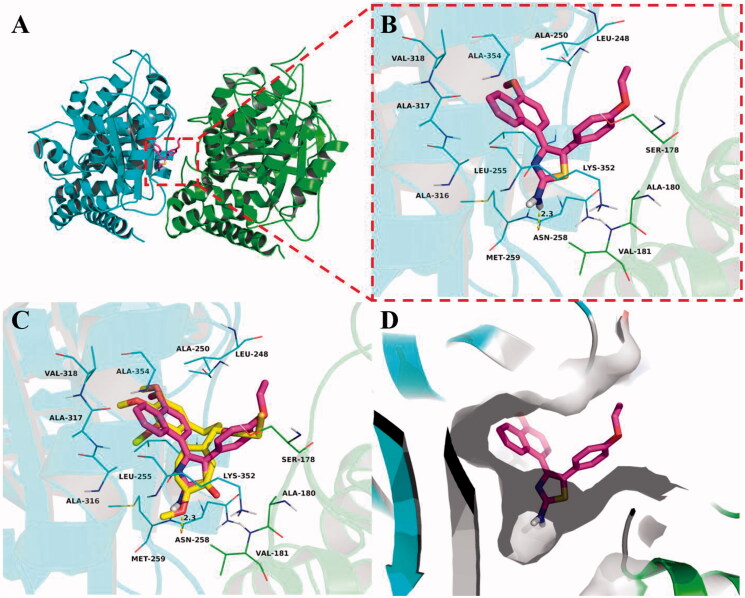
Compound **5b** was docked to the binding pocket of the α,β-tubulin (α: green; β: cyan). (A) Overall structure of α,β-tubulin with **5b**. (B) Binding pose of **5b** at colchicine binding site. (C) Superimposed pose of **5b** (pink) and colchicine (yellow) in the binding site. (D) Binding pose of **5b** in the surface of colchicine binding pocket.

Molecular docking studies were carried out to elucidate the binding mode of this series of compounds with the colchicine binding site of tubulin. To verify the accuracy of the docking results, colchicine was first docked into the colchicine binding site of tubulin. The co-crystallized conformation of colchicine was reproduced approximately (RMSD: 1.083 Å), indicating that the protocol of molecular docking can reproduce the crystallographic pose of colchicine. Then, we investigate the theoretical binding mode of compound **5b** with tubulin. As shown in Figure 6, **5b** adopted an “L-shaped” conformation in the pocket of the tubulin with estimated binding energy of −9.1 kcal·mol^−1^. The 4-methoxynaphthalene moiety of **5b** located at the hydrophobic pocket, surrounded by the residues A/Ala-180, A/Val-181, B/Leu-248, B/Ala-250, B/Leu-255, B/Met-259, B/Ala-316, B/Ala-317, B/Val-318 and B/Ala-354, forming a strong hydrophobic binding. Detailed analysis showed that the phenyl group of **5b** formed a cation-π interaction with the residue B/Lys-352. It was shown that the residue B/Asn-258 (bond length: 2.3 Å) formed a hydrogen bond with **5b**, which was the main interaction between **5b** and tubulin. All these interactions helped **5b** to anchor in the binding site of the tubulin.

### 3.6. Molecular dynamics (MD) simulations

**Figure 7.**
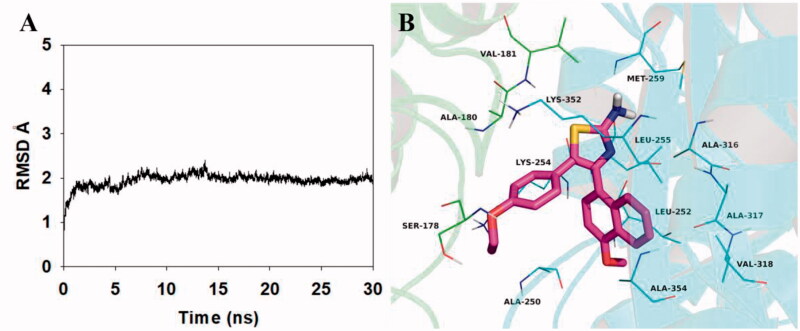
Molecular docking and molecular dynamics refinement of compound **5b** with tubulin. (A) The root-mean-square deviation (RMSD) of all the atoms of tubulin-**5b** complex with respect to its initial structure as function of time. (B) Molecular dynamics results of tubulin-**5b** complex.

To explore the potential binding mode between **5b** and the α,β-tubulin, molecular docking and molecular dynamics simulations were performed using the AutoDock vina 1.1.2 and Amber 12 software package. The preferential binding mechanism of α,β-tubulin with **5b** was determined by 30-ns molecular dynamics simulations based on the docking results. To explore the dynamic stability of the models and to ensure the rationality of the sampling strategy, the root-mean-square deviation (RMSD) value of the protein backbone based on the starting structure along the simulation time was calculated and plotted in Figure 7(A). As shown in Figure 7(A), the protein structure of the system was stabilised during the simulation.

The theoretical binding mode between **5b** and α,β-tubulin was shown in Figure 7(B). Compound **5b** adopted a compact conformation in the pocket of the α,β-tubulin. The compound **5b** located at the hydrophobic pocket, surrounded by the residues A/Ala-180, A/Val-181, B/Ala-250, B/Leu-252, B/Leu-255, B/Met-259, B/Ala-316, B/Ala-317, B/Val-318 and B/Ala-354, forming a strong hydrophobic binding. Detailed analysis showed that the phenyl group of **5b** formed cation-π interactions with the residues Lys-254 and Lys-352. All these interactions helped **5b** to anchor in the binding site of the α,β-tubulin. All in all, the above molecular dynamics simulation give us rational explanation of the interaction between **5b** and the α,β-tubulin, which provided valuable information for further development of α,β-tubulin inhibitors.

## 4. Conclusion

In summary, a novel series of thiazole-naphthalene derivatives (**5a–5c** and **6a–6n**) have been designed, synthesised, and characterised by various analytical techniques such as HRMS, ^1^H NMR, and ^13 ^C NMR. Antiproliferative activity of these newly prepared compounds was evaluated on the human breast cancer cell line (MCF-7) and human lung adenocarcinoma cell line (A549) using the CCK-8 assay. Amongst all the tested compounds, compound **5b** containing an ethoxy group at the 4-position of the phenyl ring and free amine group at the thiazole ring was found to be the most active compound with IC_50_ values of 0.48 ± 0.03 and 0.97 ± 0.13 μM. Mechanistic studies revealed that **5b** significantly inhibited tubulin polymerisation with an IC_50_ value of 3.3 µM. Cellular mechanism studies elucidated that **5b** arrested the cell cycle at G2/M phase and induced apoptosis in MCF-7 cancer cells. Furthermore, molecular docking study indicated that **5b** binds well to the colchicine binding site of tubulin.

## Supplementary Material

Supplemental Material

## References

[1] Jordan MA, Wilson L. Microtubules as a target for anticancer drugs. Nat Rev Cancer 2004;4:253–65. 15057285 10.1038/nrc1317

[2] Sorger PK, Dobles M, Tournebize R, et al. Coupling cell division and cell death to microtubule dynamics. Curr Opin Cell Biol 1997;9:807–14. 9425345 10.1016/s0955-0674(97)80081-6

[3] Downing KH, Nogales E. Tubulin structure: insights into microtubule properties and functions. Curr Opin Struct Biol 1998;8:785–91. 9914260 10.1016/s0959-440x(98)80099-7

[4] Schwartz EL. Antivascular actions of microtubule-binding drugs. Clin Cancer Res 2009;15:2594–601. 19351751 10.1158/1078-0432.CCR-08-2710 PMC2745203

[5] Amos LA. Microtubule structure and its stabilisation. Org Biomol Chem 2004;2:2153–60. 15280946 10.1039/b403634d

[6] Pasquier E, André N, Braguer D. Targeting microtubules to inhibit angiogenesis and disrupt tumour vasculature: Implications for cancer treatment. Curr Cancer Drug Targets 2007;7:566–81. 17896922 10.2174/156800907781662266

[7] Shao Y-Y, Yin Y, Lian B-P, et al. Synthesis and biological evaluation of novel shikonin-benzo[b]furan derivatives as tubulin polymerization inhibitors targeting the colchicine binding site. Eur J Med Chem 2020;190:112105. 32035399 10.1016/j.ejmech.2020.112105

[8] Sigalapalli DK, Pooladanda V, Singh P, et al. Discovery of certain benzyl/phenethyl thiazolidinone-indole hybrids as potential anti-proliferative agents: Synthesis, molecular modeling and tubulin polymerization inhibition study. Bioorg Chem 2019;92:103188. 31450167 10.1016/j.bioorg.2019.103188

[9] Stanton RA, Gernert KM, Nettles JH, et al. Drugs that target dynamic microtubules: a new molecular perspective. Med Res Rev 2011;31:443–81. 21381049 10.1002/med.20242 PMC3155728

[10] Dumontet C, Jordan MA. Microtubule-binding agents: a dynamic field of cancer therapeutics. Nat Rev Drug Discov 2010;9:790–803. 20885410 10.1038/nrd3253 PMC3194401

[11] Kaur R, Kaur G, Gill RK, et al. Recent developments in tubulin polymerization inhibitors: an overview. Eur J Med Chem 2014;87:89–124. 25240869 10.1016/j.ejmech.2014.09.051

[12] Gümüş M, Yakan M, Koca İ. Recent advances of thiazole hybrids in biological applications. Future Med Chem 2019;11:1979–98. 31517529 10.4155/fmc-2018-0196

[13] Mishra R, Sharma PK, Verma PK, et al. Biological potential of thiazole derivatives of synthetic origin. J Heterocycl Chem 2017;54:2103–16.

[14] Chhabria MT, Patel S, Modi P, et al. Thiazole: a review on chemistry, synthesis and therapeutic importance of its derivatives. Curr Top Med Chem 2016;16:2841–62. 27150376 10.2174/1568026616666160506130731

[15] Sharma PC, Bansal KK, Sharma A, et al. Thiazole-containing compounds as therapeutic targets for cancer therapy. Eur J Med Chem 2020;188:112016. 31926469 10.1016/j.ejmech.2019.112016

[16] Ayati A, Emami S, Moghimi S, et al. Thiazole in the targeted anticancer drug discovery. Future Med Chem 2019;11:1929–52. 31313595 10.4155/fmc-2018-0416

[17] Jain S, Pattnaik S, Pathak K, et al. Anticancer potential of thiazole derivatives: a retrospective review. Mini Rev Med Chem 2018;18:640–55. 29173166 10.2174/1389557517666171123211321

[18] Ohsumi K, Hatanaka T, Fujita K, et al. Syntheses and antitumor activity of cis-restricted combretastatins: 5-membered heterocyclic analogues. Bioorg Med Chem Lett 1998;8:3153–8. 9873694 10.1016/s0960-894x(98)00579-4

[19] Sun ML, Xu QL, Xu JW, et al. Synthesis and bioevaluation of *N*,4-diaryl-1,3-thiazole-2-amines as tubulin inhibitors with potent antiproliferative activity. PLoS One 2017;12:e0174006. 28333984 10.1371/journal.pone.0174006 PMC5363846

[20] Shaik TB, Hussaini SMA, Nayak VL, et al. Rational design and synthesis of 2-anilinopyridinyl-benzothiazole schiff bases as antimitotic agents. Bioorg Med Chem Lett 2017;27:2549–58. 28400235 10.1016/j.bmcl.2017.03.089

[21] Salehi M, Amini M, Ostad SN, et al. Synthesis, cytotoxic evaluation and molecular docking study of 2-alkylthio-4-(2,3,4-trimethoxyphenyl)-5-aryl-thiazoles as tubulin polymerization inhibitors. Bioorg Med Chem 2013;21:7648–54. 24238904 10.1016/j.bmc.2013.10.030

[22] Wang G, Peng Z, Zhang J, et al. Synthesis, biological evaluation and molecular docking studies of aminochalcone derivatives as potential anticancer agents by targeting tubulin colchicine binding site. Bioorg Chem 2018;78:332–40. 29627654 10.1016/j.bioorg.2018.03.028

[23] Wang G, Liu W, Gong Z, et al. Synthesis, biological evaluation, and molecular modelling of new naphthalene-chalcone derivatives as potential anticancer agents on MCF-7 breast cancer cells by targeting tubulin colchicine binding site. J Enzyme Inhib Med Chem 2020;35:139–44. 31724435 10.1080/14756366.2019.1690479 PMC6882462

[24] Wang G, Liu W, Tang J, et al. Design, synthesis, and anticancer evaluation of benzophenone derivatives bearing naphthalene moiety as novel tubulin polymerization inhibitors. Bioorg Chem 2020;104:104265. 32919128 10.1016/j.bioorg.2020.104265

[25] Maya ABS, Perez-Melero C, Mateo C, et al. Further naphthylcombretastatins. An investigation on the role of the naphthalene moiety. J Med Chem 2005;48:556–68. 15658869 10.1021/jm0310737

[26] Wang G, Qiu J, Xiao X, et al. Synthesis, biological evaluation and molecular docking studies of a new series of chalcones containing naphthalene moiety as anticancer agents. Bioorg Chem 2018;76:249–57. 29197743 10.1016/j.bioorg.2017.11.017

[27] Viegas-Junior C, Danuello A, VdS B, et al. Molecular hybridization: a useful tool in the design of new drug prototypes. Curr Med Chem 2007;14:1829–52. 17627520 10.2174/092986707781058805

[28] Ivasiv V, Albertini C, Goncalves AE, et al. Molecular hybridization as a tool for designing multitarget drug candidates for complex diseases. Curr Top Med Chem 2019;19:1694–711. 31237210 10.2174/1568026619666190619115735

[29] Wang XD, Wei W, Wang PF, et al. Novel 3-arylfuran-2(5h)-one-fluoroquinolone hybrid: design, synthesis and evaluation as antibacterial agent. Bioorg Med Chem 2014;22:3620–8. 24882676 10.1016/j.bmc.2014.05.018

[30] Xiao ZP, Wang XD, Wang PF, et al. Design, synthesis, and evaluation of novel fluoroquinolone-flavonoid hybrids as potent antibiotics against drug-resistant microorganisms. Eur J Med Chem 2014;80:92–100. 24769347 10.1016/j.ejmech.2014.04.037

[31] Hao S-Y, Qi Z-Y, Wang S, et al. Synthesis and bioevaluation of n-(3,4,5-trimethoxyphenyl)-1h-pyrazolo[3,4-b]pyridin-3-amines as tubulin polymerization inhibitors with anti-angiogenic effects. Bioorg Med Chem 2021;31:115985. 33421913 10.1016/j.bmc.2020.115985

[32] Mustafa M, Anwar S, Elgamal F, et al. Potent combretastatin a-4 analogs containing 1,2,4-triazole: Synthesis, antiproliferative, anti-tubulin activity, and docking study. Eur J Med Chem 2019;183:111697. 31536891 10.1016/j.ejmech.2019.111697

