# Supplementary material for: Design, synthesis and biological evaluation of novel thiazole-naphthalene derivatives as potential anticancer agents and tubulin polymerisation inhibitors
Source: J Enzyme Inhib Med Chem. 2021 Jul 26;36(1):1694–702. doi: 10.1080/14756366.2021.1958213 (PMC8317958; doi:10.1080/14756366.2021.1958213)
Supplement: Supplemental Material [file IENZ_A_1958213_SM1728.pdf]

Supplemental Material

1. Experimental section.

1.1. Chemistry.

All starting materials and reagents were purchased from commercial suppliers. Nuclear magnetic resonance spectra (NMR) were recorded on a JNM spectrometer (400 MHz) with TMS as an external reference and reported in parts per million. High-resolution mass spectra (HRMS) were recorded on Bruker MicroQTOF II and Shimadzu LCMS-IT-TOF using ESI method.

1.1.1. General procedure for the synthesis of 5

A mixture of **4** (10 mmol) and thiourea (10 mmol) in EtOH (100 mL) was stirred at reflux for 3 h. After the completion of the reaction, the solvent was removed under reduced pressure and the residue was purified by silica gel column chromatography (petroleum ether/EtOAc) to give compounds **5a**-**5c**, respectively.

4-(4-methoxynaphthalen-1-yl)-5-(4-methoxyphenyl)thiazol-2-amine (**5a**)

^{1}H NMR ($CDCl_3$, 400 MHz) δ: 3.68 (s, 3H), 3.99 (s, 3H), 5.59 (s, 2H), 6.59 (d, 2H, J = 8.8 Hz), 6.72 (d, 1H, J = 8.0 Hz), 6.94 (d, 2H, J = 8.8 Hz), 7.29 (d, 1H, J = 8.0 Hz), 7.42-7.45 (m, 2H), 7.82-7.85 (m, 1H), 8.26-8.29 (m, 1H); ^{13}C NMR ($CDCl_3$, 100 MHz) δ: 55.30, 55.84, 103.78, 114.38, 117.53, 120.65, 121.38, 122.69, 124.41, 125.86, 126.04, 127.33, 128.03, 129.16, 130.01, 131.79, 141.14, 146.16, 157.38, 159.82, 167.76; HRMS (ESI) calcd for $[M+H]^+$ $C_{21}H_{19}N_2O_2S^+$: 363.1162 found 363.1126.

5-(4-ethoxyphenyl)-4-(4-methoxynaphthalen-1-yl)thiazol-2-amine (**5b**)

^{1}H NMR ($CDCl_3$, 400 MHz) δ: 1.31 (t, 3H, J = 6.8 Hz), 3.85 (q, 2H, J = 6.8 Hz), 4.00 (s, 3H), 6.30 (s, 2H), 6.56 (d, 2H, J = 8.8 Hz), 6.74 (d, 1H, J = 8.0 Hz), 6.88 (d, 2H, J = 8.0 Hz), 7.31 (d, 1H, J = 8.0 Hz), 7.37-7.46 (m, 2H), 7.76-7.78 (m, 1H), 8.26-8.28 (m, 1H); ^{13}C NMR ($CDCl_3$, 100 MHz) δ: 14.81, 21.13, 55.57, 63.39, 103.58, 114.42, 122.13, 122.92, 123.88, 124.10, 125.35, 125.71, 125.83, 126.90, 128.74, 129.27, 132.88, 141.49, 155.90, 157.98, 166.36, 176.67; HRMS (ESI) calcd for $[M+H]^+$ $C_{22}H_{20}N_2O_2S^+$: 377.1318 found 377.1302.

5-(2-bromo-3,4,5-trimethoxyphenyl)-4-(4-methoxynaphthalen-1-yl)thiazol-2-amine

(**5c**)

^{1}H NMR (d_6-DMSO, 400 MHz) δ: 3.36 (s, 3H), 3.67 (s, 6H), 3.92 (s, 3H), 6.62 (s, 1H), 6.84 (d, 1H, J = 8.0 Hz), 7.17 (s, 2H), 7.24 (d, 1H, J = 8.0 Hz), 7.45-7.46 (m, 2H), 7.97-7.99 (m, 1H), 8.11-8.13 (m, 1H); ^{13}C NMR ($CDCl_3$, 100 MHz) δ: 56.0, 56.1, 61.1, 61.2, 104.1, 111.4, 113.0, 119.4, 121.9, 125.2, 125.6, 126.0, 126.6, 126.9, 128.6, 129.5, 133.0, 142.5, 148.0, 150.7, 152.2, 155.0, 167.4; HRMS (ESI) calcd for $[M+H]^+$ $C_{23}H_{21}BrN_2O_4S^+$: 501.0478 found 501.0426.

1.1.2. General procedure for the synthesis of 6

A mixture of **5** (1 mmol) and corresponding acid anhydride (1 mL) was stirred at 120 °C for 3 h. After the completion of the reaction, the mixture was poured into 50 mL of water and extracted with ethyl acetate. The combined organic layers were dried over Na_2SO_4 and concentrated under vacuum. The residue was purified by chromatography to give the title compounds **6**.

N-(4-(4-methoxynaphthalen-1-yl)-5-(4-methoxyphenyl)thiazol-2-yl)acetamide (**6a**)

^{1}H NMR ($CDCl_3$, 400 MHz) δ: 1.48 (s, 3H), 3.72 (s, 3H), 4.03 (s, 3H), 6.66 (d, 2H, J = 8.8 Hz), 6.76 (d, 1H, J = 8.0 Hz), 7.07 (d, 2H, J = 8.8 Hz), 7.33 (d, 1H, J = 8.0 Hz), 7.38-7.48 (m, 2H), 7.90 (d, 1H, J = 8.0 Hz), 8.30-8.32 (m, 1H), 11.47 (s, 1H); ^{13}C NMR ($CDCl_3$, 100 MHz) δ: 21.8, 55.3, 55.7, 103.5, 114.1, 122.3, 124.2, 124.6, 125.6, 127.0, 128.4, 129.1, 129.7, 132.9, 141.8, 156.0, 156.9, 158.9, 168.6; HRMS (ESI) calcd for $[M+H]^+$ $C_{23}H_{21}N_2O_3S^+$: 405.1267 found 405.1234.

N-(4-(4-methoxynaphthalen-1-yl)-5-(4-methoxyphenyl)thiazol-2-yl)propionamide (**6b**)

^{1}H NMR ($CDCl_3$, 400 MHz) δ: 0.85 (t, 3H, J = 6.8 Hz), 1.77 (q, 2H, J = 6.8 Hz), 3.71 (s, 3H), 4.03 (s, 3H), 6.65 (d, 2H, J = 8.8 Hz), 6.77 (d, 1H, J = 8.0 Hz), 7.07 (d, 2H, J = 8.8 Hz), 7.34 (d, 1H, J = 8.0 Hz), 7.35-7.48 (m, 2H), 7.75 (d, 1H, J = 8.0 Hz), 8.30 (d, 1H, J = 8.0 Hz), 11.20 (s, 1H); ^{13}C NMR ($CDCl_3$, 100 MHz) δ: 8.6, 28.4, 55.2, 55.7, 103.5, 114.1, 122.3, 124.2, 124.6, 125.6, 125.9, 127.0, 128.3, 129.1, 129.7, 132.9, 141.6, 156.0, 156.9, 158.9, 172.4; HRMS (ESI) calcd for $[M+H]^+$ $C_{24}H_{23}N_2O_3S^+$: 419.1424 found 419.1392.

N-(4-(4-methoxynaphthalen-1-yl)-5-(4-methoxyphenyl)thiazol-2-yl)butyramide (**6c**)

^{1}H NMR ($CDCl_3$, 400 MHz) δ: 0.66 (t, 3H, J = 6.8 Hz),, 1.34 (sext, 2H, J = 6.8 Hz),,

1.69 (q, 2H, J = 6.8 Hz), 3.72 (s, 3H), 4.03 (s, 3H), 6.66 (d, 2H, J = 8.8 Hz), 6.77 (d, 1H, J = 8.0 Hz), 7.07 (d, 2H, J = 8.8 Hz), 7.36 (d, 1H, J = 8.0 Hz), 7.37-7.47 (m, 2H), 7.79 (d, 1H, J = 8.0 Hz), 8.30 (d, 1H, J = 8.0 Hz), 11.38 (s, 1H); ^{13}C NMR ($CDCl_3$, 100 MHz) δ: 13.3, 18.0, 36.8, 55.3, 55.6, 103.6, 114.1, 122.3, 124.3, 124.5, 125.6, 125.9, 127.0, 128.3, 129.1, 129.7, 132.8, 141.6, 156.0, 156.9, 158.9, 171.1; HRMS (ESI) calcd for $[M+H]^+$ $C_{25}H_{25}N_2O_3S^+$: 433.1580 found 433.1542.

2,2,2-trichloro-*N*-(4-(4-methoxynaphthalen-1-yl)-5-(4-methoxyphenyl)thiazol-2-yl)acetamide (**6d**)

^{1}H NMR ($CDCl_3$, 400 MHz) δ: 3.69 (s, 3H), 4.02 (s, 3H), 6.62 (d, 2H, J = 8.8 Hz), 6.82 (d, 1H, J = 8.0 Hz), 6.91 (d, 2H, J = 8.8 Hz), 7.41-7.49 (m, 3H), 7.60 (d, 1H, J = 8.0 Hz), 8.28 (d, 1H, J = 8.0 Hz), 9.06 (s, 1H); ^{13}C NMR ($CDCl_3$, 100 MHz) δ: 55.5, 56.1, 79.7, `104.5, 114.5, 121.2, 122.2, 125.4, 125.9, 126.3, 127.2, 129.1, 129.3, 133.0, 155.4, 158.4, 165.9; HRMS (ESI) calcd for $[M+H]^+$ $C^{23}H^{18}C^{l3}N^2O^3S^+$: 507.0098 found 507.0096.

N-(4-(4-methoxynaphthalen-1-yl)-5-(4-methoxyphenyl)thiazol-2-yl)isobutyramide (**6e**)

^{1}H NMR ($CDCl_3$, 400 MHz) δ: 0.81 (d, 6H, J = 6.8 Hz), 1.81 (hept, 1H, J = 6.8 Hz), 3.69 (s, 3H), 4.01 (s, 3H), 6.63 (d, 2H, J = 8.8 Hz), 6.76 (d, 1H, J = 8.0 Hz), 7.05 (d, 2H, J = 8.8 Hz), 7.35-7.44 (m, 3H), 7.77 (d, 1H, J = 8.0 Hz), 8.28 (d, 1H, J = 8.0 Hz), 10.83 (s, 1H); ^{13}C NMR ($CDCl_3$, 100 MHz) δ: 19.0, 34.9, 55.2, 55.7, 103.6, 114.0, 122.3, 124.4, 125.0, 125.5, 125.6, 126.0, 127.0, 128.6, 128.9, 129.7, 132.9, 142.3, 155.9, 156.4, 158.8, 175.5; HRMS (ESI) calcd for $[M+H]^+$ $C_{25}H_{25}N_2O_3S^+$: 433.1580 found 433.1543.

N-(5-(4-ethoxyphenyl)-4-(4-methoxynaphthalen-1-yl)thiazol-2-yl)acetamide (**6f**)

^{1}H NMR ($CDCl_3$, 400 MHz) δ: 1.33 (t, 3H, J = 6.8 Hz), 1.54 (s, 3H), 3.89 (q, 2H, J = 6.8 Hz), 4.01 (s, 3H), 6.63 (d, 2H, J = 8.8 Hz), 6.74 (d, 1H, J = 8.0 Hz), 7.04 (d, 2H, J = 8.8 Hz), 7.31 (d, 1H, J = 8.0 Hz), 7.36-7.40 (m, 1H), 7.43-7.47 (m, 1H), 7.77 (d, 1H, J = 8.0 Hz), 8.28 (d, 1H, J = 8.0 Hz), 11.51 (s, 1H); ^{13}C NMR ($CDCl_3$, 100 MHz) δ: 14.83, 22.18, 55.66, 63.43, 103.52, 114.59, 122.34, 123.88, 124.24, 125.50, 125.60, 125.91, 127.00, 128.55, 129.11, 129.73, 132.93, 141.31, 156.05, 156.71, 158.39, 168.52; HRMS (ESI) calcd for $[M+H]^+$ $C_{24}H_{23}N_2O_3S^+$: 419.1424 found 419.1383.

N-(5-(4-ethoxyphenyl)-4-(4-methoxynaphthalen-1-yl)thiazol-2-yl)propionamide (**6g**)

^{1}H NMR ($CDCl_3$, 400 MHz) δ: 0.83 (t, 3H, J = 6.8 Hz), 1.35 (t, 3H, J = 6.8 Hz), 1.73(q, 2H, J = 6.8 Hz), 3.90 (q, 2H, J = 6.8 Hz), 4.03 (s, 3H), 6.63 (d, 2H, J = 8.0 Hz), 6.77 (d, 1H, J = 8.0 Hz), 7.06 (d, 2H, J = 8.0 Hz), 7.34 (d, 1H, J = 8.0 Hz), 7.38-7.48 (m, 2H), 7.79 (d, 1H, J = 8.0 Hz), 8.29 (d, 1H, J = 8.0 Hz), 10.93 (s, 1H); ^{13}C NMR ($CDCl_3$, 100 MHz) δ: 8.83, 14.84, 28.81, 55.63, 63.40, 103.53, 114.53, 122.24, 124.25, 125.09, 125.46, 125.65, 125.90, 126.93, 128.65, 128.91, 129.69, 133.02, 142.35, 155.85, 156.14, 158.23, 172.05; HRMS (ESI) calcd for $[M+H]^+$ $C_{25}H_{25}N_2O_3S^+$: 433.1580 found 433.1550;

N-(5-(4-ethoxyphenyl)-4-(4-methoxynaphthalen-1-yl)thiazol-2-yl)butyramide (**6h**)

^{1}H NMR ($CDCl_3$, 400 MHz) δ: 0.66 (t, 3H, J = 6.8 Hz), 1.33-1.41 (m, 5H), 1.68 (t, 3H, J = 6.8 Hz), 3.90 (q, 2H, J = 6.8 Hz), 4.03 (s, 3H), 6.63 (d, 2H, J = 8.0 Hz), 6.77 (d, 1H, J = 8.0 Hz), 7.05 (d, 2H, J = 8.0 Hz), 7.36 (d, 1H, J = 8.0 Hz), 7.38-7.47 (m, 2H), 7.80 (d, 1H, J = 8.0 Hz), 8.29 (d, 1H, J = 8.0 Hz), 11.26 (s, 1H); ^{13}C NMR ($CDCl_3$, 100 MHz) δ: 13.3, 14.9, 17.9, 36.8, 55.6, 63.4, 103.6, 114.5, 122.3, 124.1, 124.6, 125.6, 125.9, 127.0, 128.4, 129.1, 129.7, 132.8, 141.7, 155.9, 156.9, 158.3, 171.6; HRMS (ESI) calcd for $[M+H]^+$ $C_{26}H_{27}N_2O_3S^+$: 447.1737 found 447.1702.

N-(5-(4-ethoxyphenyl)-4-(4-methoxynaphthalen-1-yl)thiazol-2-yl)isobutyramide (**6i**)

^{1}H NMR ($CDCl_3$, 400 MHz) δ: 0.94 (d, 6H, J = 6.8 Hz), 1.35 (t, 3H, J = 6.8 Hz), 2.01-2.07 (hept, 1H, J = 6.8 Hz), 3.90 (q, 2H, J = 6.8 Hz), 4.03 (s, 3H), 6.64 (d, 2H, J = 8.8 Hz), 6.78 (d, 1H, J = 8.0 Hz), 7.06 (d, 2H, J = 8.8 Hz), 7.36 (d, 1H, J = 8.0 Hz), 7.40-7.49 (m, 2H), 7.77 (d, 1H, J = 8.0 Hz), 8.30 (d, 1H, J = 8.0 Hz), 10.71 (s, 1H); ^{13}C NMR ($CDCl_3$, 100 MHz) δ: 14.8, 19.0, 34.8, 55.7, 63.4, 103.6, 114.5, 122.3, 124.1, 124.7, 125.5, 126.0, 127.0, 128.6, 129.0, 129.6, 132.8, 141.8, 156.0, 156.7, 158.3, 175.6; HRMS (ESI) calcd for $[M+H]^+$ $C_{26}H_{27}N_2O_3S^+$: 447.1737 found 447.1704.

N-(5-(2-bromo-3,4,5-trimethoxyphenyl)-4-(4-methoxynaphthalen-1-yl)thiazol-2-yl)propionamide (**6j**)

^{1}H NMR ($CDCl_3$, 400 MHz) δ: 0.92 (t, 3H, J = 6.8 Hz), 1.91 (q, 2H, J = 6.8 Hz), 3.38 (s, 3H), 3.81 (s, 3H), 3.82 (s, 3H), 3.99 (s, 3H), 6.42 (s, 1H), 6.70 (d, 1H, J = 8.0 Hz), 7.26-7.27 (m, 1H), 7.30 (d, 1H, J = 8.0 Hz), 7.42-7.44 (m, 2H), 7.86-7.88 (m, 1H), 8.24-

8.26 (m, 1H), 11.43 (s, 1H); ^{13}C NMR ($CDCl_3$, 100 MHz) δ: 8.73, 28.90, 55.65, 55.88, 61.06, 61.16, 103.24, 111.68, 111.74, 122.32, 123.19, 125.39, 125.46, 125.69, 126.77, 126.91, 127.66, 128.93, 132.86, 143.21, 144.33, 151.19, 152.26, 156.19, 158.61, 172.31; HRMS (ESI) calcd for $[M+H]^+$ $C_{26}H_{26}BrN_2O_5S^+$: 557.0740 found 557.0730.

N-(5-(2-bromo-3,4,5-trimethoxyphenyl)-4-(4-methoxynaphthalen-1-yl)thiazol-2-yl)isobutyramide (**6k**)

^{1}H NMR ($CDCl_3$, 400 MHz) δ: 0.80 (d, 6H, J = 6.8 Hz), 1.81 (hept, 1H, J = 6.8 Hz), 3.39 (s, 3H), 3.81 (s, 3H), 3.81 (s, 3H), 3.99 (s, 3H), 6.44 (s, 1H), 6.70 (d, 1H, J = 8.0 Hz), 7.30 (d, 1H, J = 8.0 Hz), 7.42-7.45 (m, 2H), 7.94-7.96 (m, 1H), 8.24-8.26 (m, 1H), 10.88 (s, 1H); ^{13}C NMR ($CDCl_3$, 100 MHz) δ: 19.0, 34.9, 55.6, 55.8, 61.1, 61.2, 103.2, 111.7, 111.8, 122.2, 124.5, 125.4, 125.6, 125.8, 126.7, 127.1, 128.4, 128.7, 133.0, 142.9, 146.1, 151.1, 152.1, 155.9, 158.2, 175.5; HRMS (ESI) calcd for $[M+H]^+$ $C_{27}H_{28}BrN_2O_5S^+$: 571.0897 found 571.0846.

2,2,2-trichloro-*N*-(5-(4-ethoxyphenyl)-4-(4-methoxynaphthalen-1-yl)thiazol-2-yl)acetamide (**6l**)

^{1}H NMR ($CDCl_3$, 400 MHz) δ: 1.33 (t, 3H, J = 6.8 Hz), 3.81 (q, 2H, J = 6.8 Hz), 4.01 (s, 3H), 6.60 (d, 2H, J = 8.8 Hz), 6.83 (d, 1H, J = 8.0 Hz), 6.88 (d, 2H, J = 8.8 Hz), 7.39-7.46 (m, 2H), 7.48 (d, 1H, J = 8.0 Hz), 7.61-7.63 (m, 1H), 8.28-8.31 (m, 1H), 9.17 (s, 1H); ^{13}C NMR ($CDCl_3$, 100 MHz) δ: 14.74, 55.78, 63.52, 95.29, 103.75, 114.80, 117.93, 120.24, 121.55, 122.58, 124.56, 125.88, 127.79, 129.10, 130.10, 131.45, 132.05, 157.27, 159.09, 166.48, 168.14; HRMS (ESI) calcd for $[M+H]^+$ $C_{24}H_{20}Cl_3N_2O_3S^+$: 521.0255 found 521.0257.

N-(5-(2-bromo-3,4,5-trimethoxyphenyl)-4-(4-methoxynaphthalen-1-yl)thiazol-2-yl)-*N*-butyrylbutyramide (**6m**)

^{1}H NMR ($CDCl_3$, 400 MHz) δ: 0.92 (t, 3H, J = 6.8 Hz), 0.95 (t, 3H, J = 6.8 Hz), 1.55 (sext, 2H, J = 6.8 Hz), 1.68 (sext, 2H, J = 6.8 Hz), 2.23 (t, 2H, J = 6.8 Hz), 2.34 (t, 2H, J = 6.8 Hz), 3.33 (s, 3H), 3.80 (s, 3H), 3.81 (s, 3H), 3.99 (s, 3H), 6.38 (s, 1H), 6.71 (d, 1H, J = 8.0 Hz), 7.28 (d, 1H, J = 8.0 Hz), 7.39-7.44 (m, 2H), 7.78-7.81 (m, 1H), 8.23-8.25 (m, 1H), 11.94 (s, 1H); ^{13}C NMR ($CDCl_3$, 100 MHz) δ: 13.7, 18.4, 36.2, 37.8, 55.6, 55.8, 61.1, 61.2, 103.2, 111.7, 122.2, 123.3, 125.3, 125.5, 126.7, 127.9, 128.8, 132.9,

143.1, 144.8, 151.1, 152.1, 156.0, 159.1, 171.8, 179.4; HRMS (ESI) calcd for $[M+H]^+$ $C_{31}H_{34}BrN_2O_6S^+$: 641.1315 found 641.1317.

N-acetyl-N-(5-(2-bromo-3,4,5-trimethoxyphenyl)-4-(4-methoxynaphthalen-1-yl)thiazol-2-yl)propionamide (**6n**)

^{1}H NMR ($CDCl_3$, 400 MHz) δ: 2.20 (s, 3H), 3.34 (s, 3H), 3.79 (s, 3H), 3.81 (s, 3H), 3.99 (s, 3H), 6.37 (s, 1H), 6.71 (d, 1H, J = 8.0 Hz), 7.28 (d, 1H, J = 8.0 Hz), 7.39-7.45 (m, 2H), 7.75-7.78 (m, 1H), 8.23-8.26 (m, 1H), 12.21 (s, 1H); ^{13}C NMR ($CDCl_3$, 100 MHz) δ: 9.0, 22.7, 27.5, 55.5, 55.8, 61.0, 61.1, 103.2, 111.6, 111.7, 122.2, 123.1, 125.3, 125.4, 125.6, 126.7, 126.7, 127.6, 128.7, 132.8, 143.1, 144.6, 151.1, 152.1, 156.0, 158.6, 168.5, 179.6; HRMS (ESI) calcd for $[M+H]^+$ $C_{28}H_{28}BrN_2O_6S^+$: 599.0846 found 599.0847.

1.2. Cell culture and cytotoxicity assay

The antiproliferative activities of the title compounds (**5a-5c** and **6a-6n**) were evaluated against MCF-7 human breast cancer cell line by using the CCK-8 assay with cisplatin, 5-fluorouracil, tamoxifen, and CA-4 as the reference drugs. The cancer cells were cultured in RPMI-1640 medium containing 10% fetal bovine serum in a humidified 5% CO_2 atmosphere at 37 °C. Cells were seeded into 96-well plates at a density of 1×10^5 cells/well and incubated for 24 h. After incubation, the cells were treated with vehicle control, reference drugs, or title compounds, and incubated at 37 °C for 48 h. Then CCK-8 was added and incubated for another 4 h. The absorbance values of the solution at 450 nm were measured using microplate reader, and each experiment was performed at least in triplicate. Then the results were expressed as the IC_{50} values.

1.3. *In vitro* tubulin polymerization assay

The purified tubulin protein was mixed with different concentrations of compound **5b** (0.8, 1.5, 3.0, 6.0, 12.5, and 25 μM), **6d** (0.8, 1.5, 3.0, 6.0, 12.5, and 25 μM), **6l** (0.8, 1.5, 3.0, 6.0, 12.5, and 25 μM), or colchicine (3.0, 6.0, 12.5, 25, 50, and 100 μM) in PEM buffer (100 mM PIPES, 1 mM $MgCl_2$, and 1 mM EGTA) with 1 mM GTP and 5% glycerol. Then the mixture was immediately monitored under 340 nm at 37 °C every 1 min for 20 min using the spectrophotometer. The plateau absorbance values were used for calculations. The IC_{50} value was defined as the concentration of the compound

that inhibited tubulin assembly by 50% after 20 min incubation.

1.4. Cell cycle analysis

MCF-7 cells were seeded in 6-well plates (3×10^5 cells/well) and incubated in the absence and presence of different concentrations of compound **5b** (0.3125, 0.625, and 1.25 μM) for 24 h. Subsequently, the cells were harvested, washed with PBS, and fixed in 70% ice-cold ethanol at 4 °C overnight. After removed the ethanol in the next day, the cells were resuspended in the staining buffer and incubated with RNase and propidium iodide for 30 min. Flow cytometry analysis was performed using flow cytometer.

1.5. Apoptosis analysis

MCF-7 cells were seeded in 6-well plates (3×10^5 cells/well) and incubated with different concentrations of compound **5b** (0.3125, 0.625, and 1.25 μM) or vehicle for 24 h. Cells were harvested and incubated with 5 μL of Annexin-V/FITC in 300 μL binding buffer for 15 min. Then, PI solution (10 μL) was added to the medium and incubated for 5 min. Finally, the percentage of apoptotic cells was analyzed using flow cytometry.

1.6. Molecular modeling

Molecular docking studies were performed to investigate the binding mode between the compound and the α,β-tubulin using Autodock vina 1.1.2. The three-dimensional (3D) coordinate of the tubulin (PDB ID: 1SA0) was downloaded from Protein Data Bank (www.rcsb.org). The 3D structure of the compounds were obtained by ChemBioDraw Ultra 14.0 and ChemBio3D Ultra 14.0 softwares. The AutoDockTools 1.5.6 package was employed to generate the docking input files. The search grid of the α,β-tubulin was identified as center_x: 118.921, center_y: 89.718, and center_z: 5.932 with dimensions size_x: 22, size_y: 22, and size_z: 22. The value of exhaustiveness was set to 16. For Vina docking, the default parameters were used if it was not mentioned. The best-scoring pose as judged by the Vina docking score was chosen and visually analyzed using PyMoL 1.7.6 software (www.pymol.org).

1.7. Molecular dynamics refinement

The Amber 12 and AmberTools 13 programs were used for MD simulations of the

selected docked pose. MBPPS was first prepared by ACPYPE, a tool based on ANTECHAMBER for generating automatic topologies and parameters in different formats for different molecular mechanics programs, including calculation of partial charges. Then, the forcefield “leaprc.gaff” (generalized amber forcefield) was used to prepare the ligand, while “leaprc.ff12SB” was used for the receptor. The system was placed in a rectangular box (with a 10.0 Å boundry) of TIP3P water using the “SolvateOct” command with the minimum distance between any solute atoms. Equilibration of the solvated complex was done by carrying out a short minimization (500 steps of each steepest descent and conjugate gradient method), 500 ps of heating, and 50 ps of density equilibration with weak restraints using the GPU (NVIDIA® Tesla K20c) accelerated PMEMD (Particle Mesh Ewald Molecular Dynamics) module. At last, 20 ns of MD simulations were carried out.

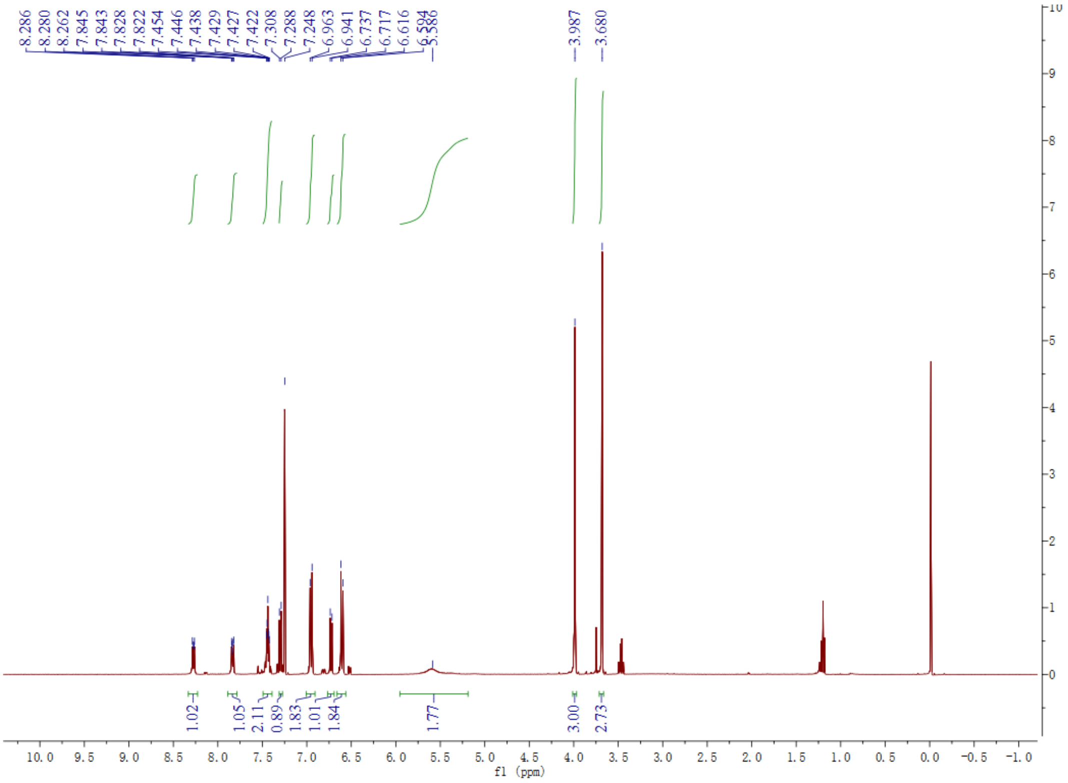

Figure S1. ^{1}H NMR of compound **5a**

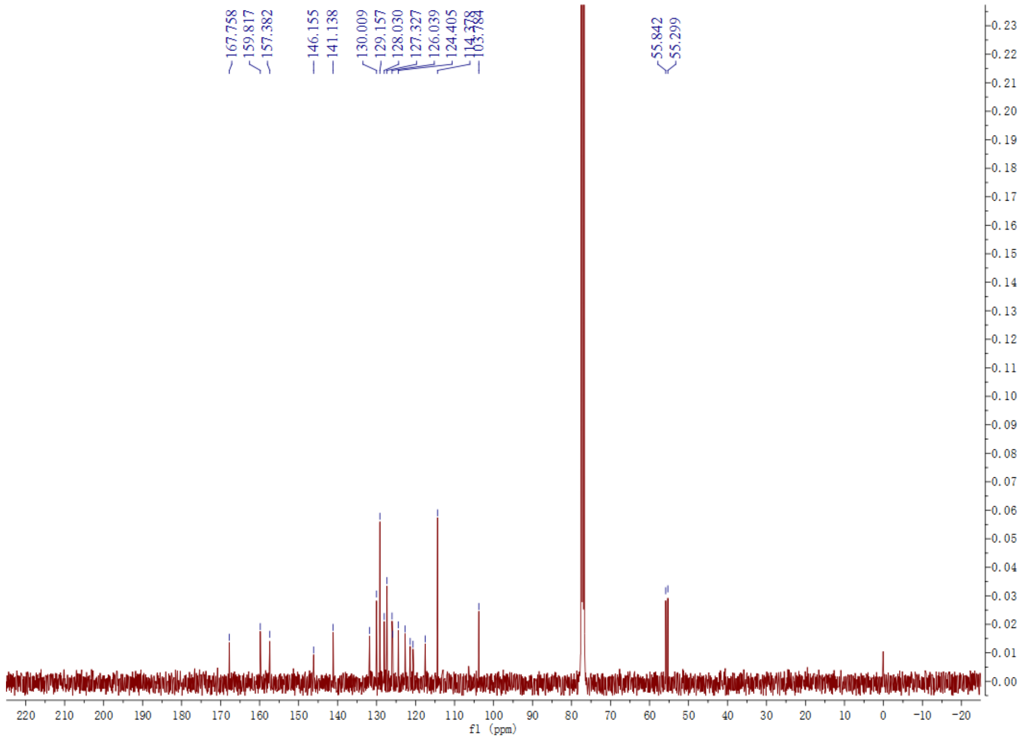

Figure S2. ^{13}C NMR of compound **5a**

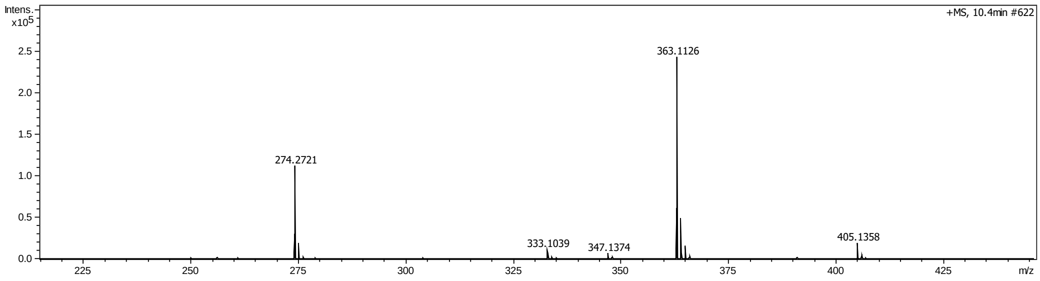

Figure S3. HRMS of compound **5a**

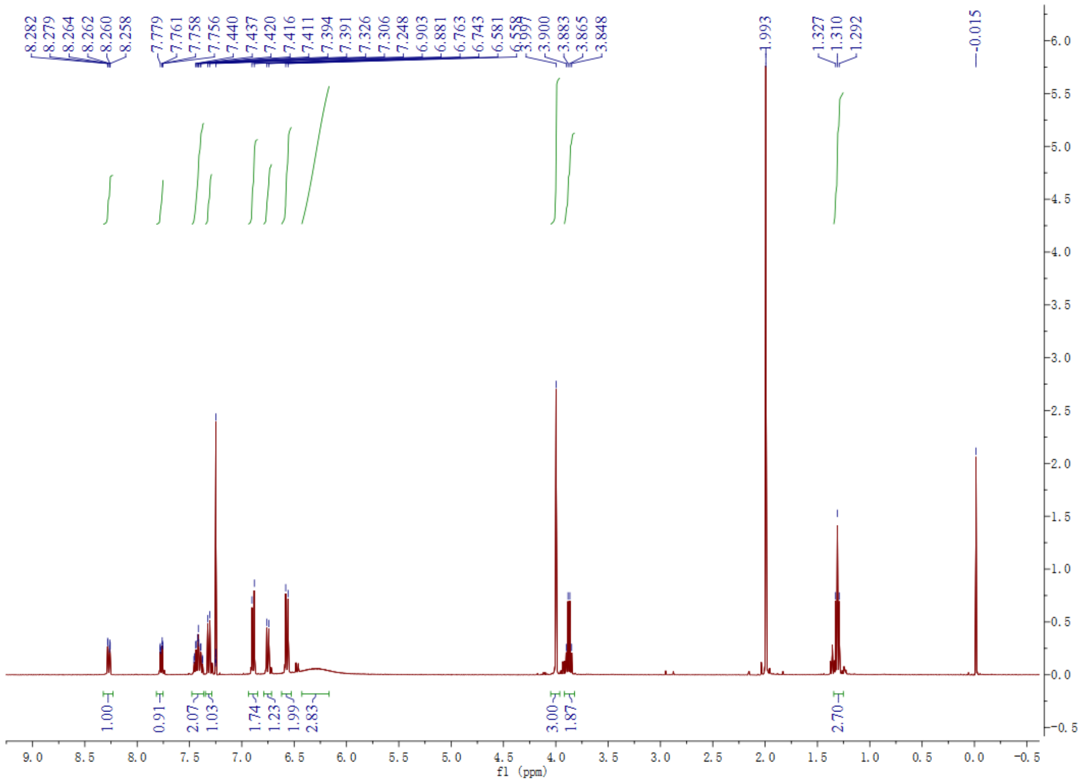

Figure S4. ^{1}H NMR of compound **5b**

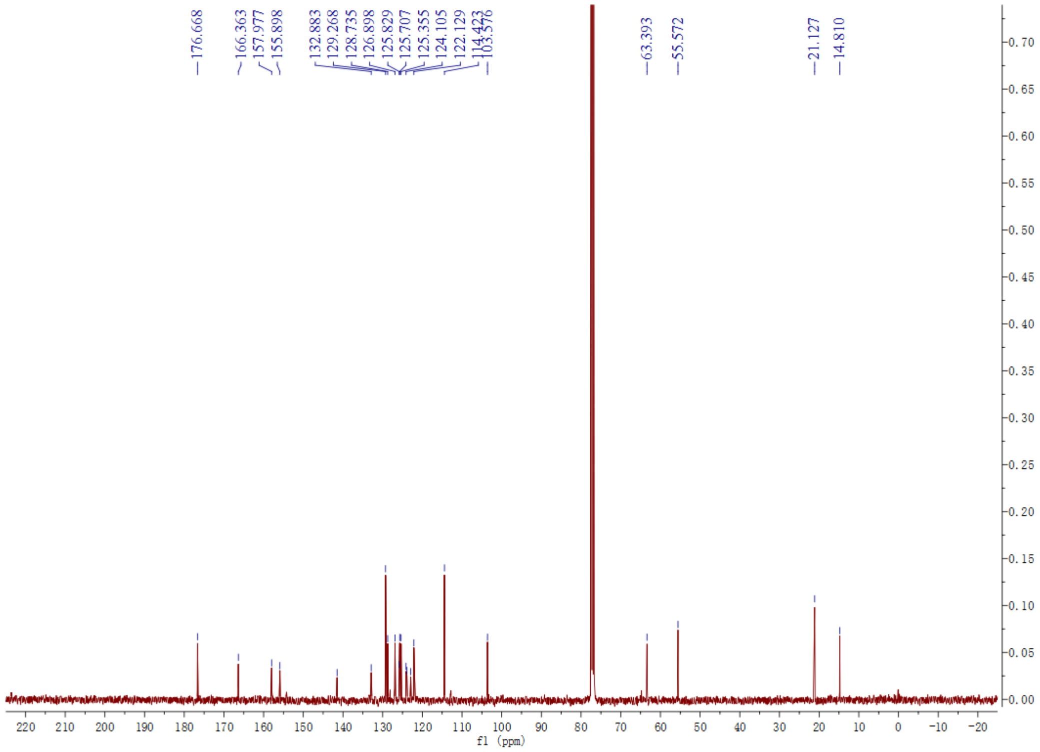

Figure S5. ^{13}C NMR of compound **5b**

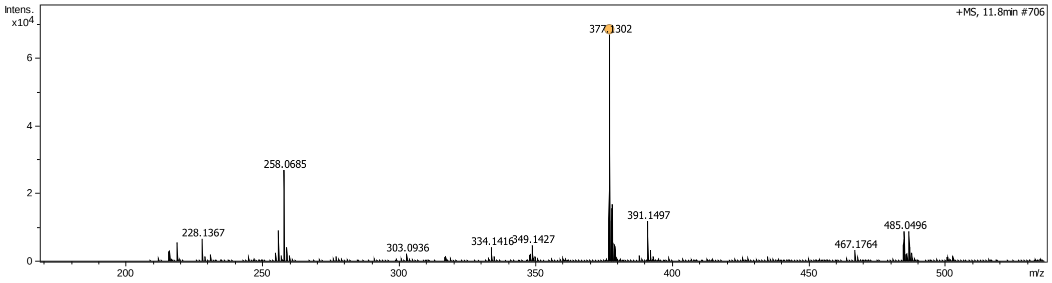

Figure S6. HRMS of compound **5b**

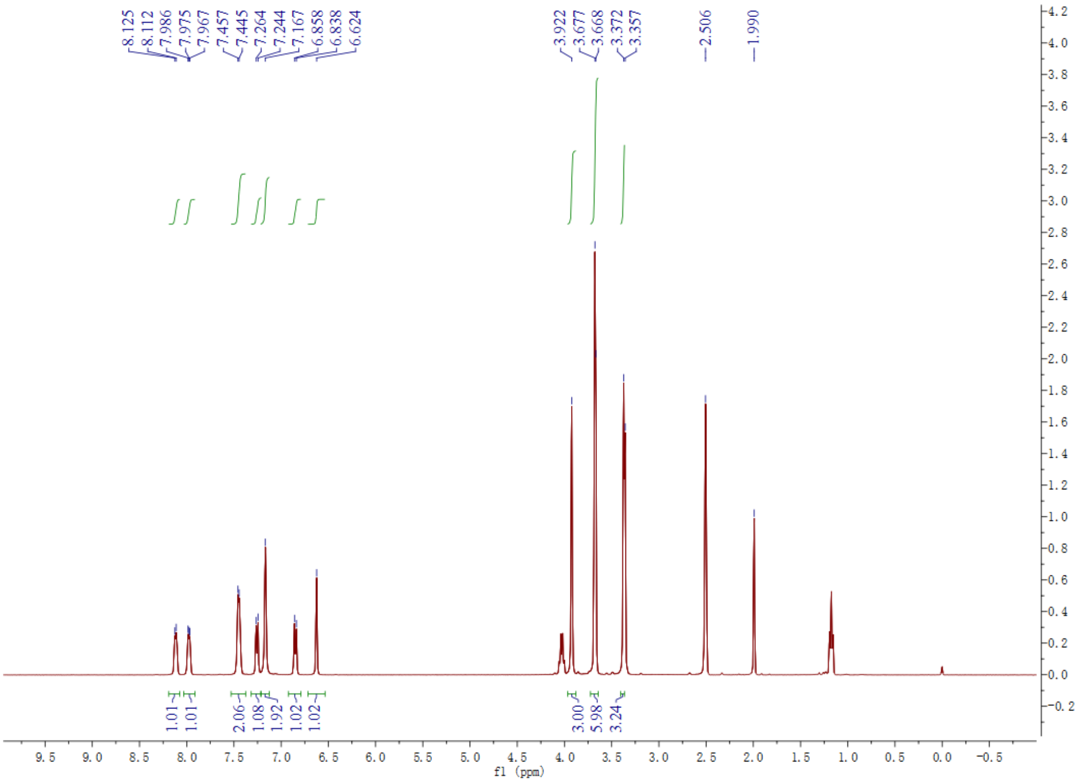

Figure S7. ^{1}H NMR of compound **5c**

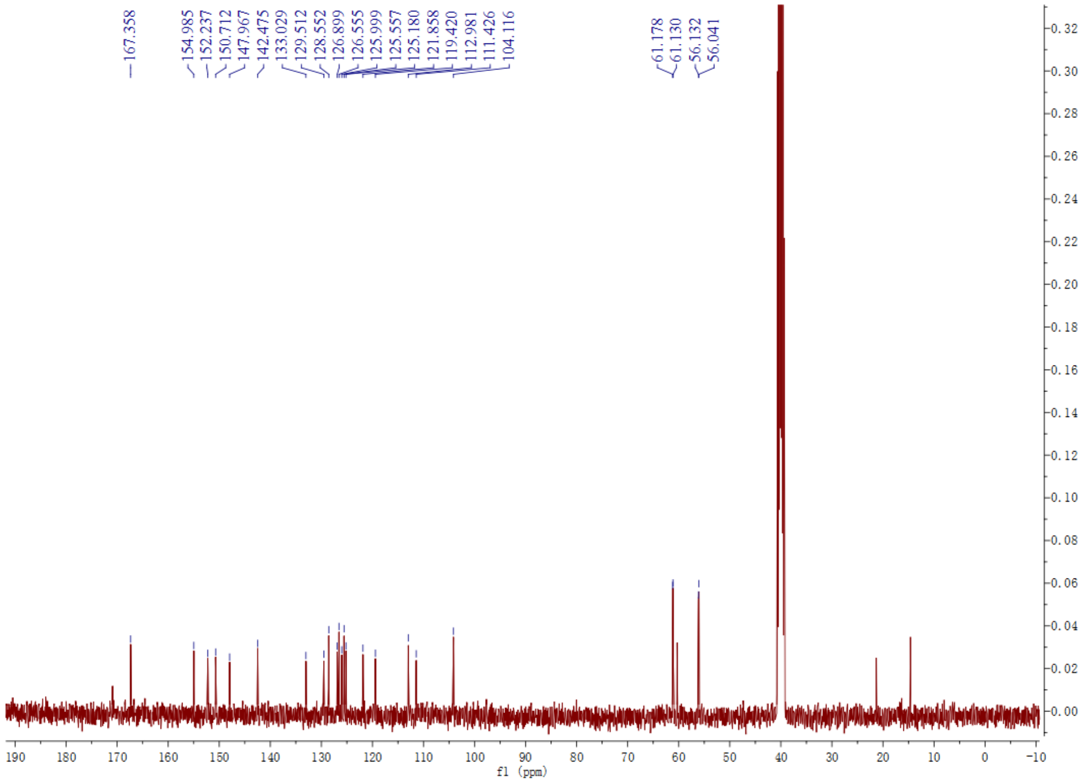

Figure S8. ^{13}C NMR of compound **5c**

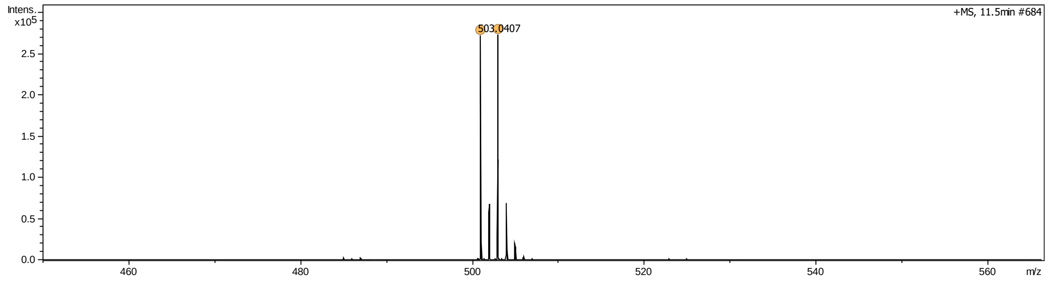

Figure S9. HRMS of compound **5c**

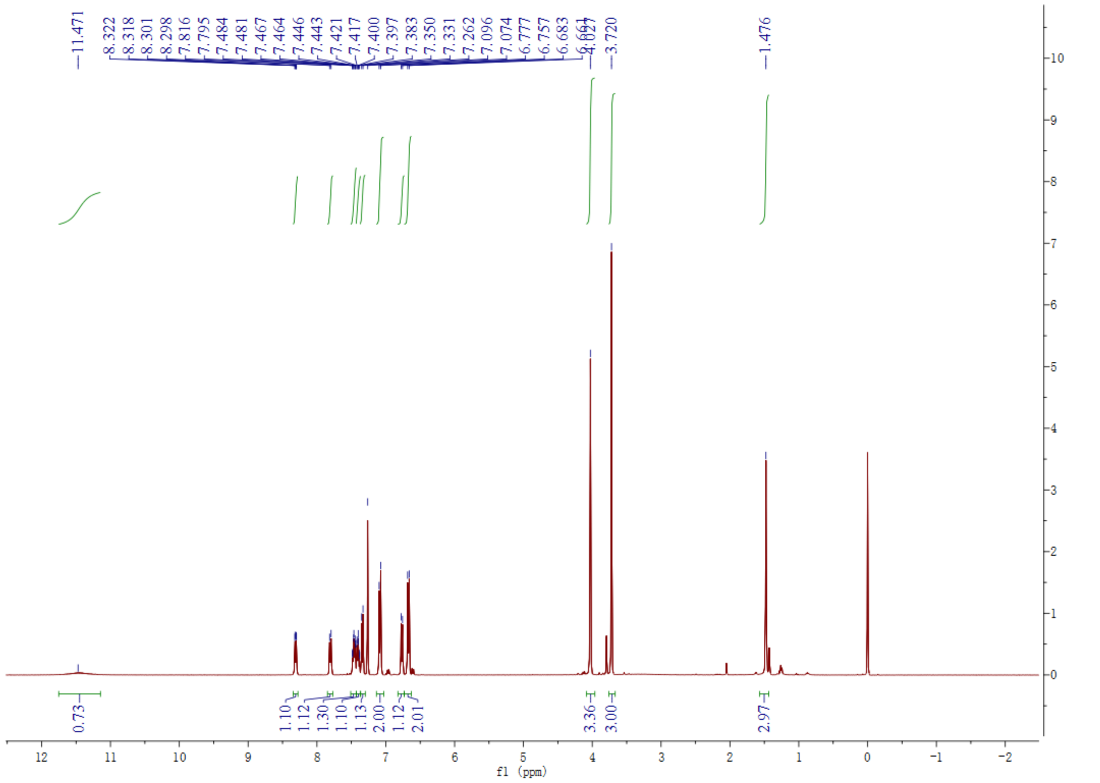

Figure S10. ^{1}H NMR of compound **6a**

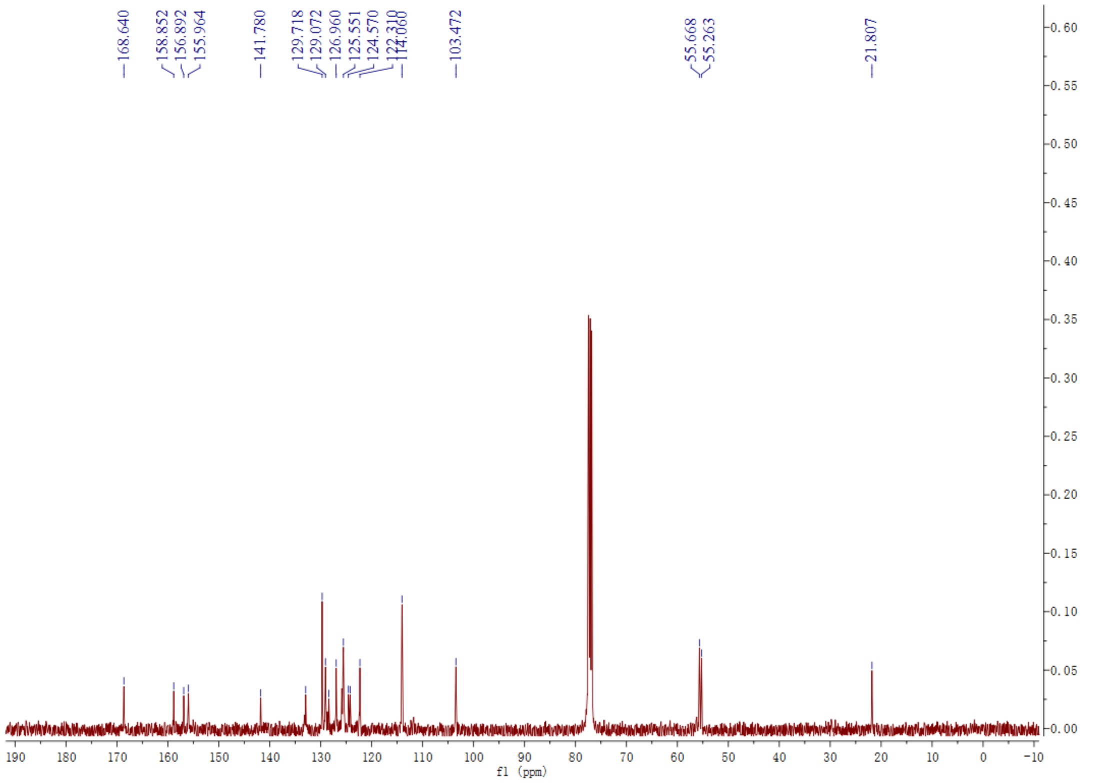

Figure S11. ^{13}C NMR of compound **6a**

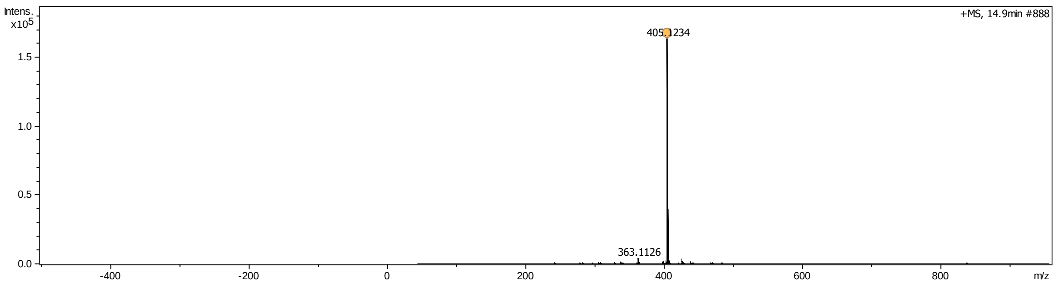

Figure S12. HRMS of compound **6a**

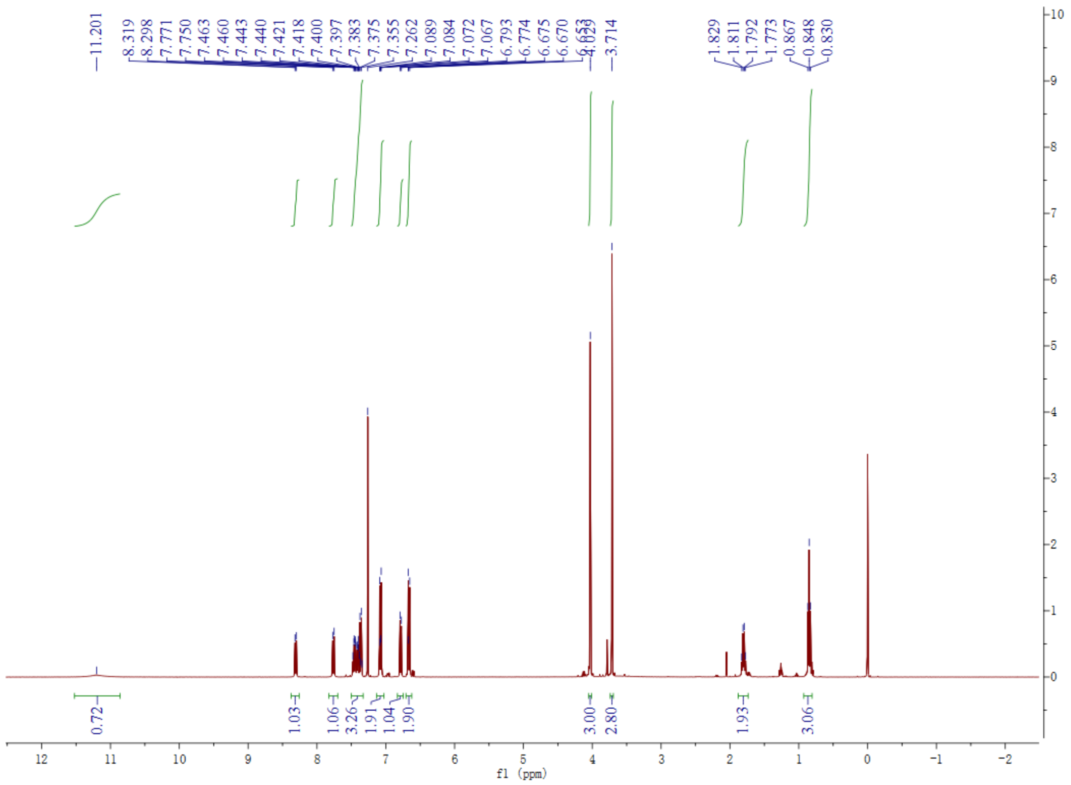

Figure S13. ^{1}H NMR of compound **6b**

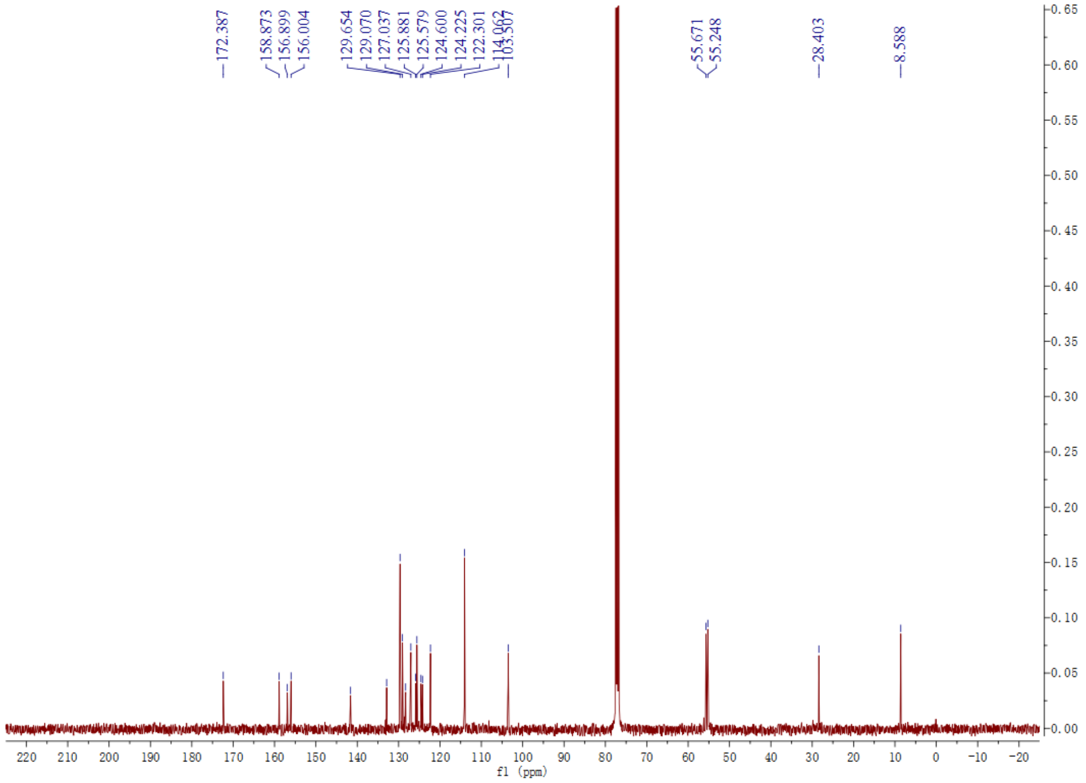

Figure S14. ^{13}C NMR of compound **6b**

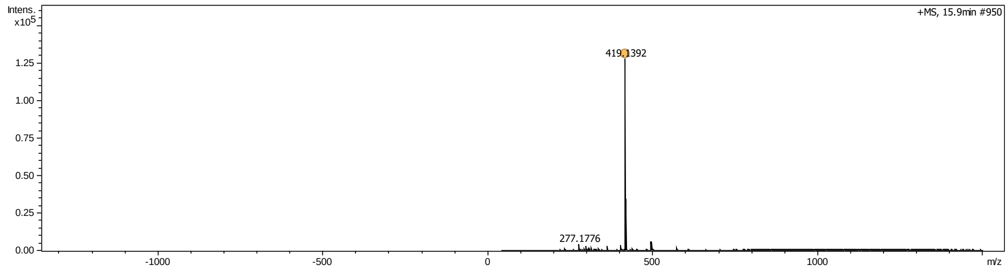

Figure S15. HRMS of compound **6b**

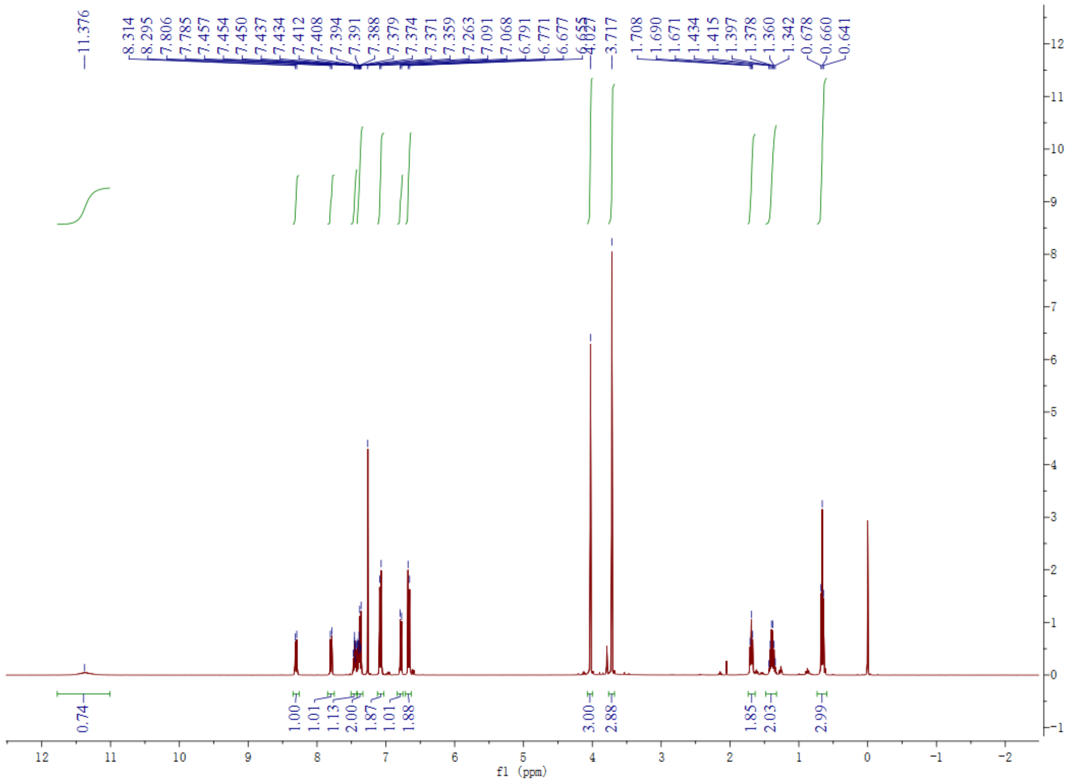

Figure S16. ^{1}H NMR of compound **6c**

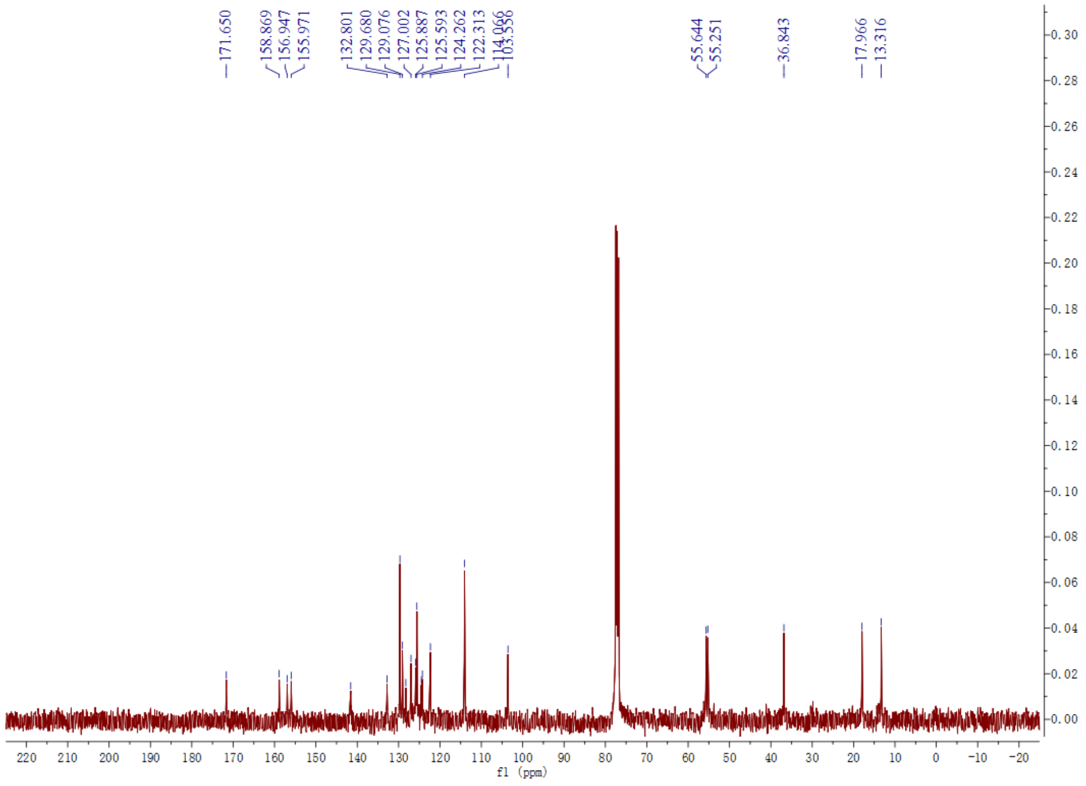

Figure S17. ^{13}C NMR of compound **6c**

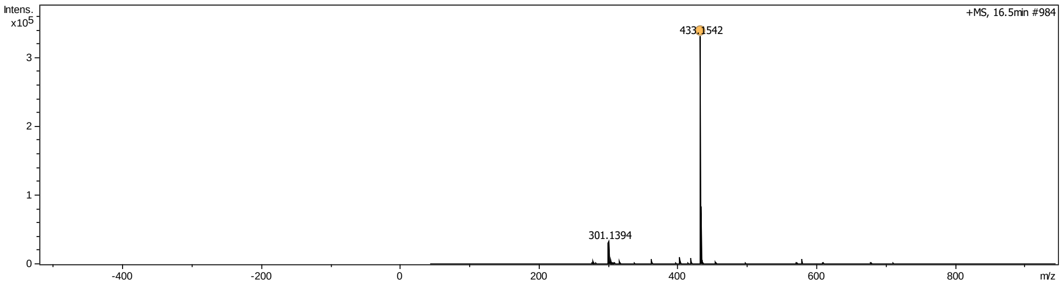

Figure S18. HRMS of compound **6c**

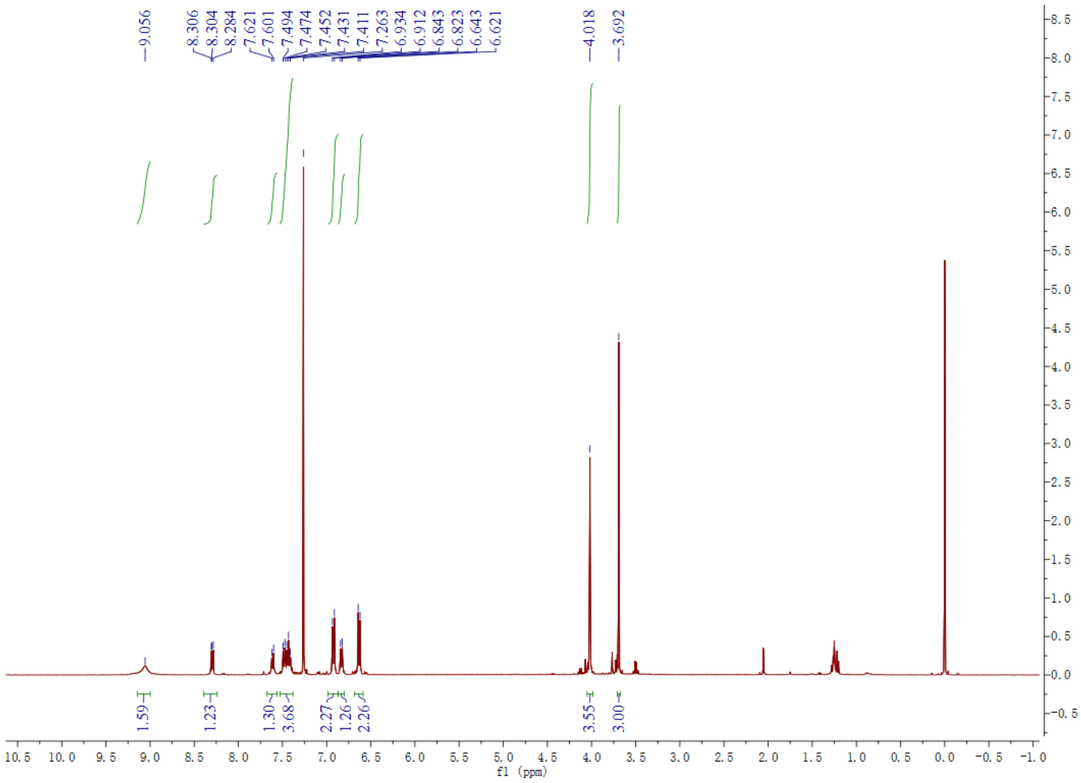

Figure S19. ^{1}H NMR of compound **6d**

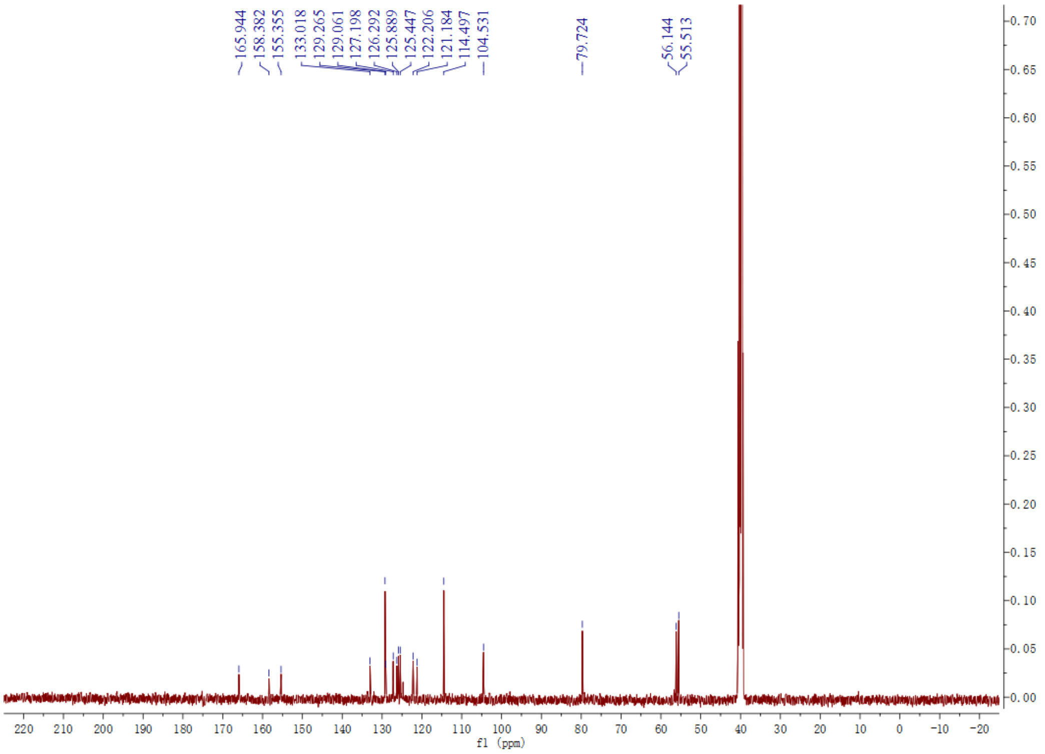

Figure S20. ^{13}C NMR of compound **6d**

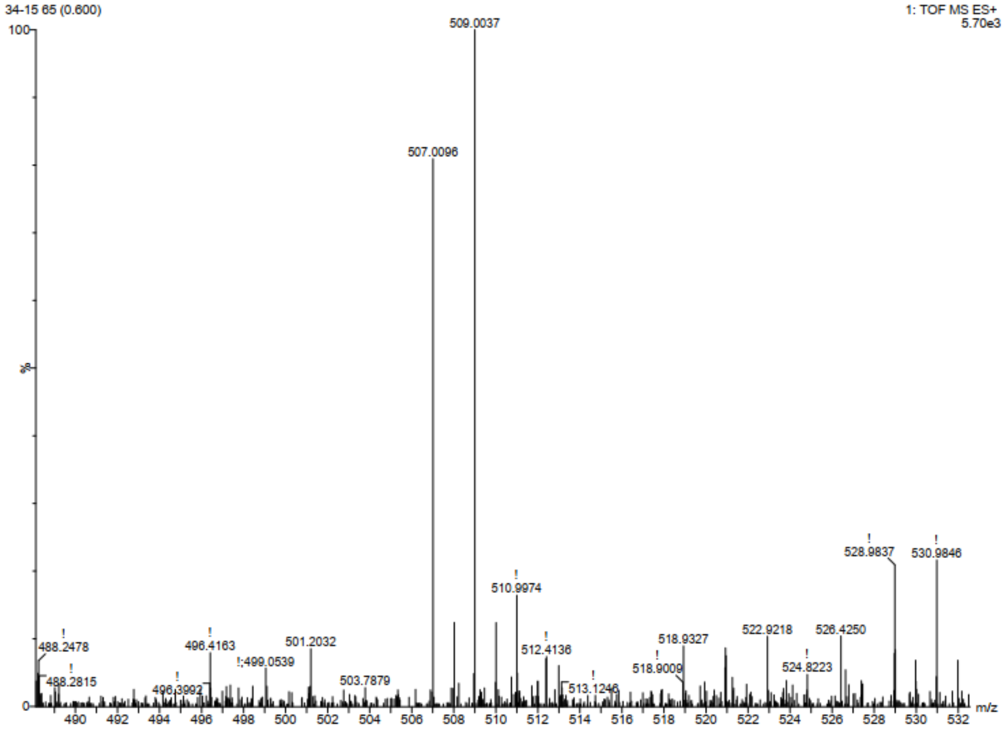

Figure S21. HRMS of compound **6d**

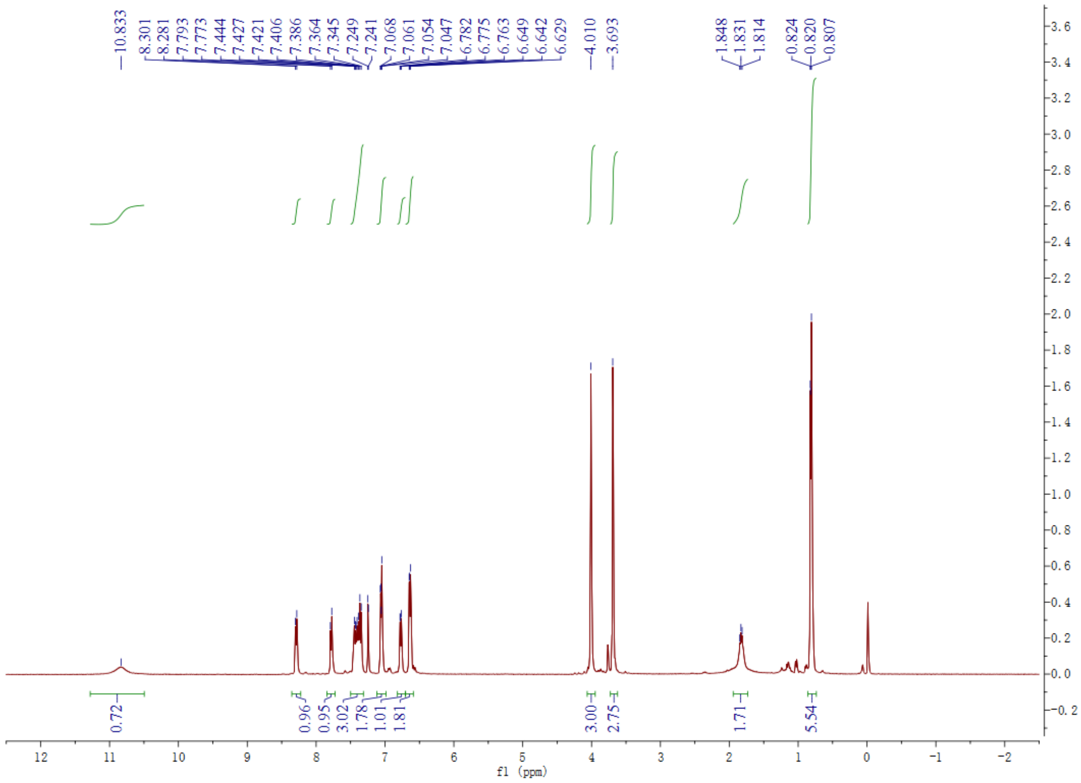

Figure S22. ^{1}H NMR of compound **6e**

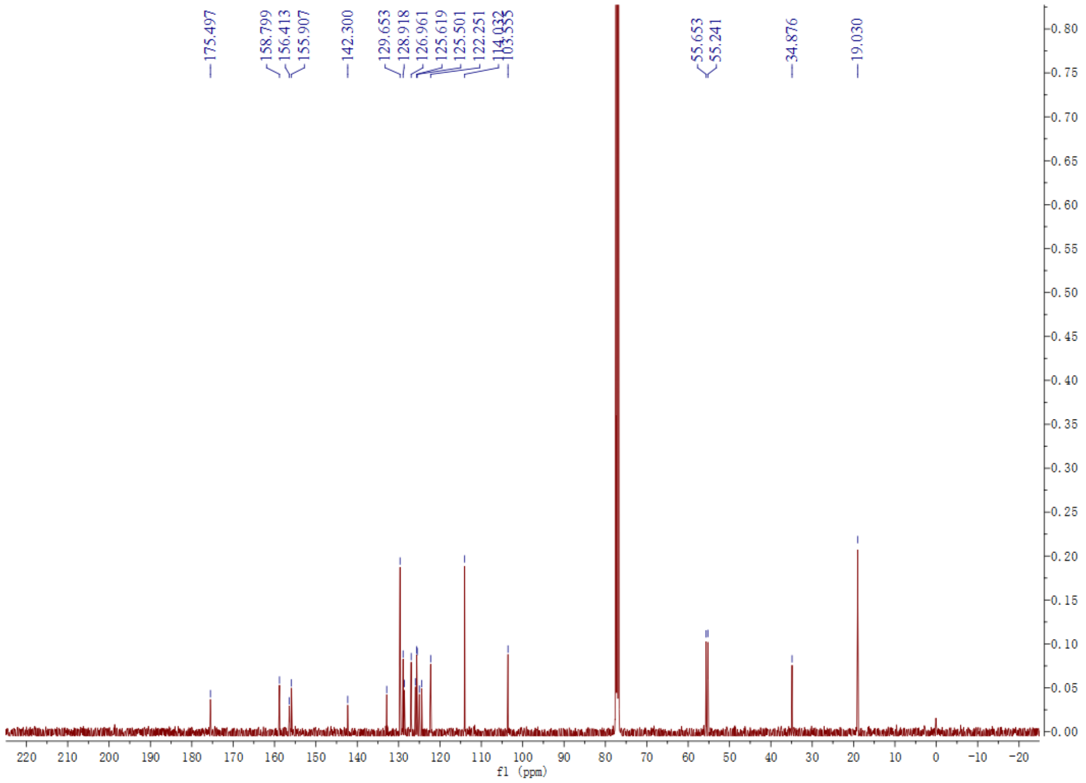

Figure S23. ^{13}C NMR of compound **6e**

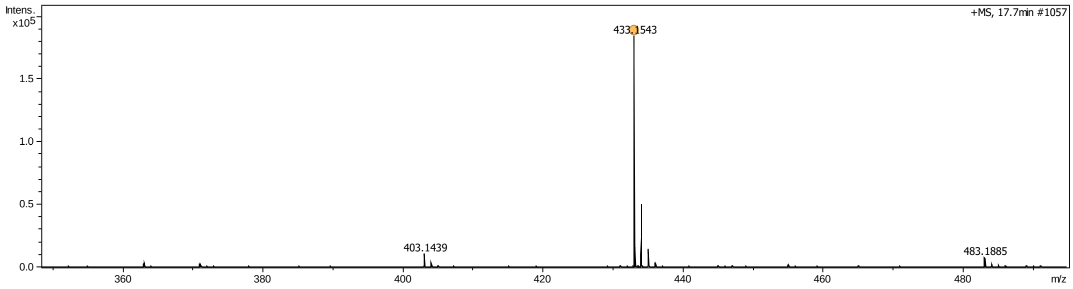

Figure S24. HRMS of compound **6e**

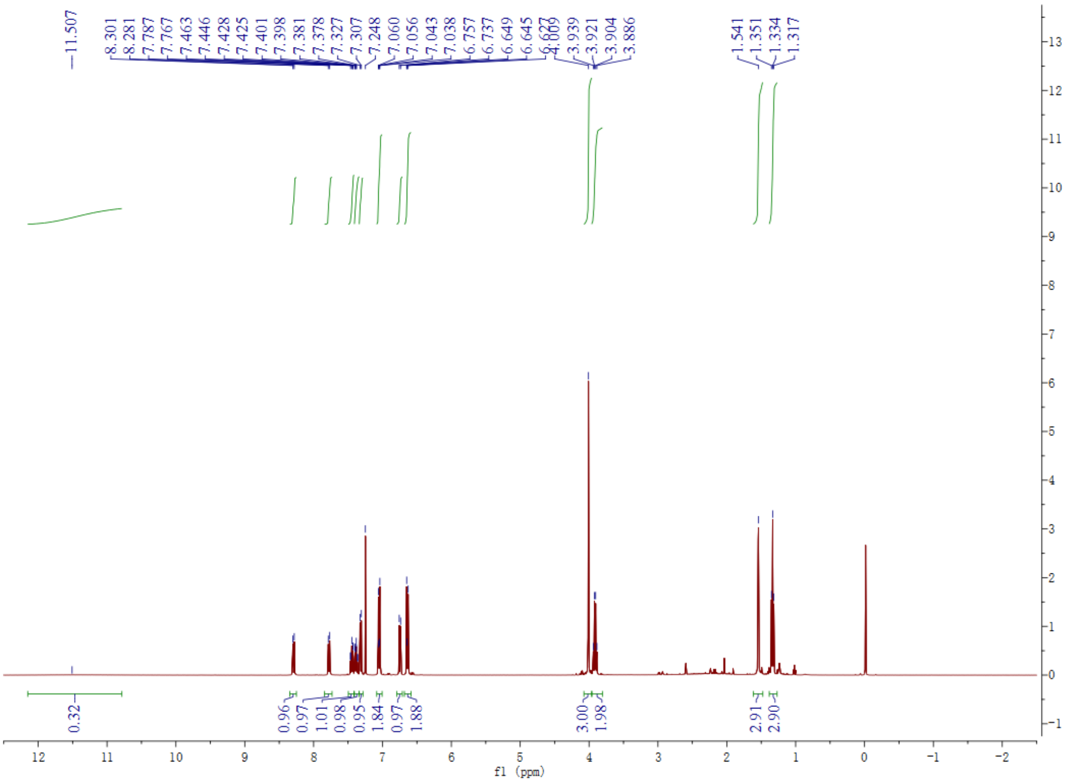

Figure S25. ^{1}H NMR of compound **6f**

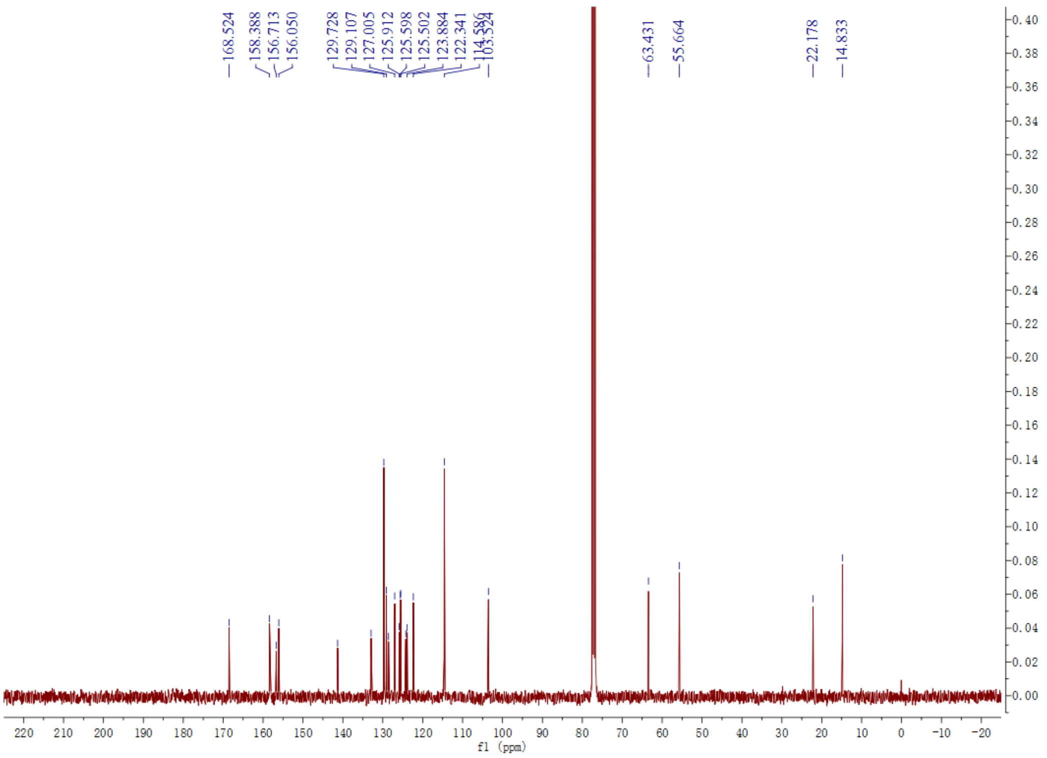

Figure S26. ^{13}C NMR of compound **6f**

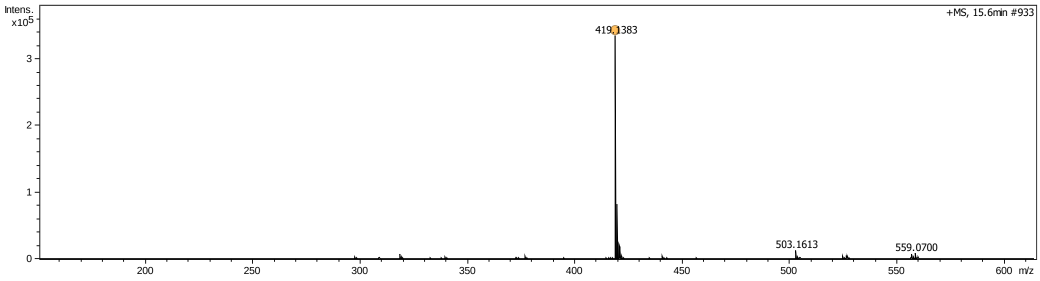

Figure S27. HRMS of compound **6f**

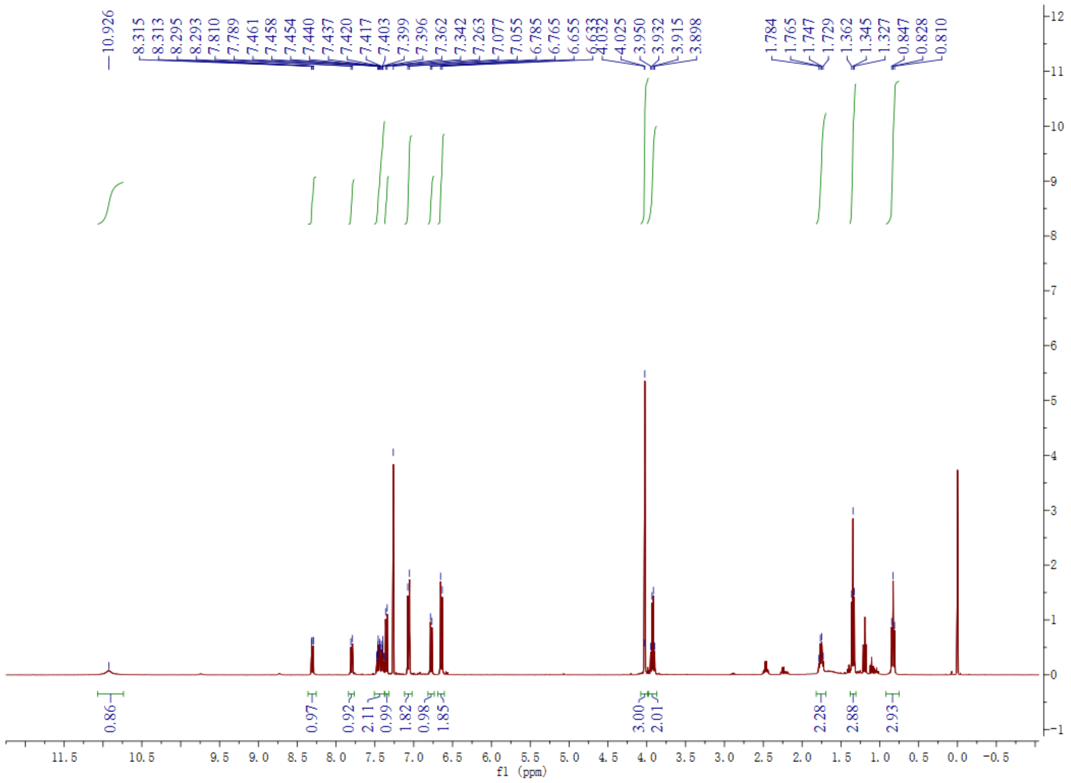

Figure S28. ^{1}H NMR of compound **6g**

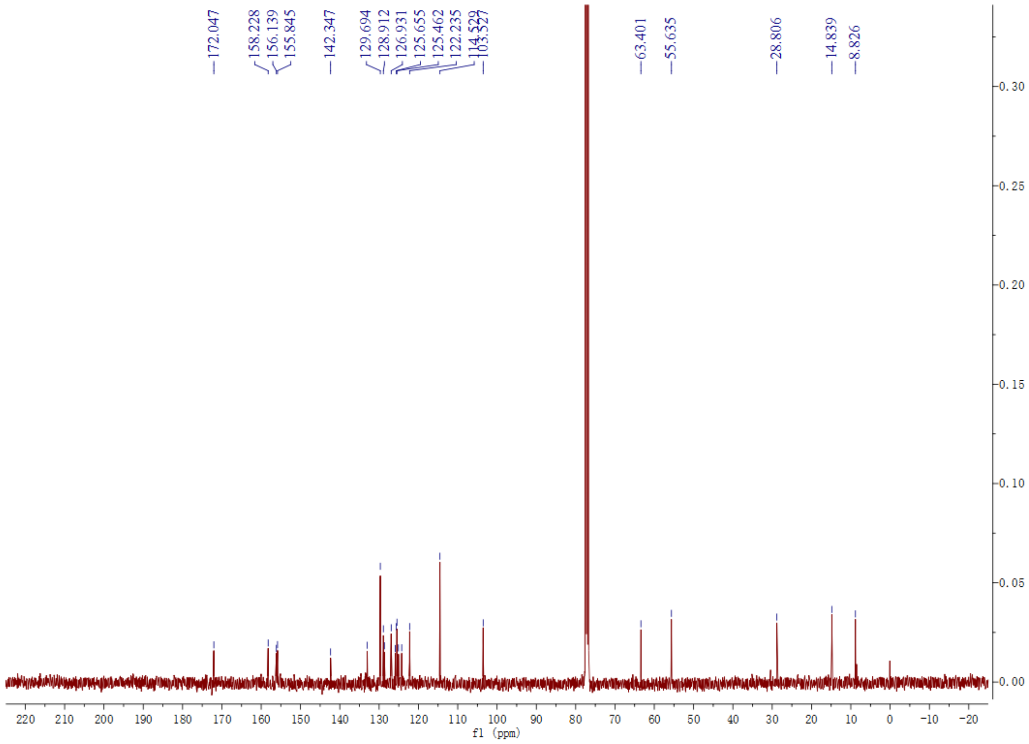

Figure S29. ^{13}C NMR of compound **6g**

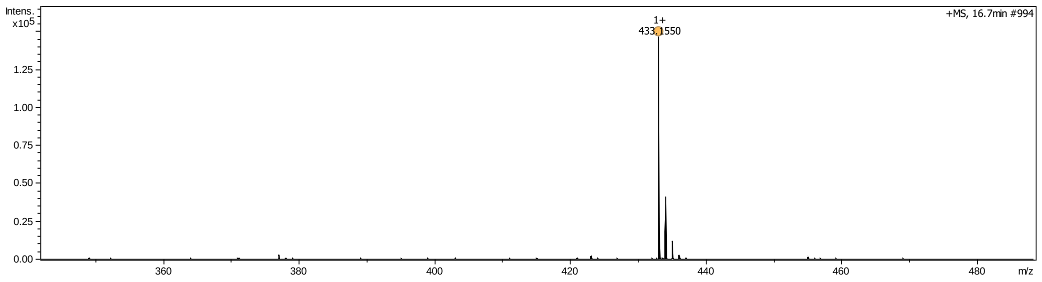

Figure S30. HRMS of compound **6g**

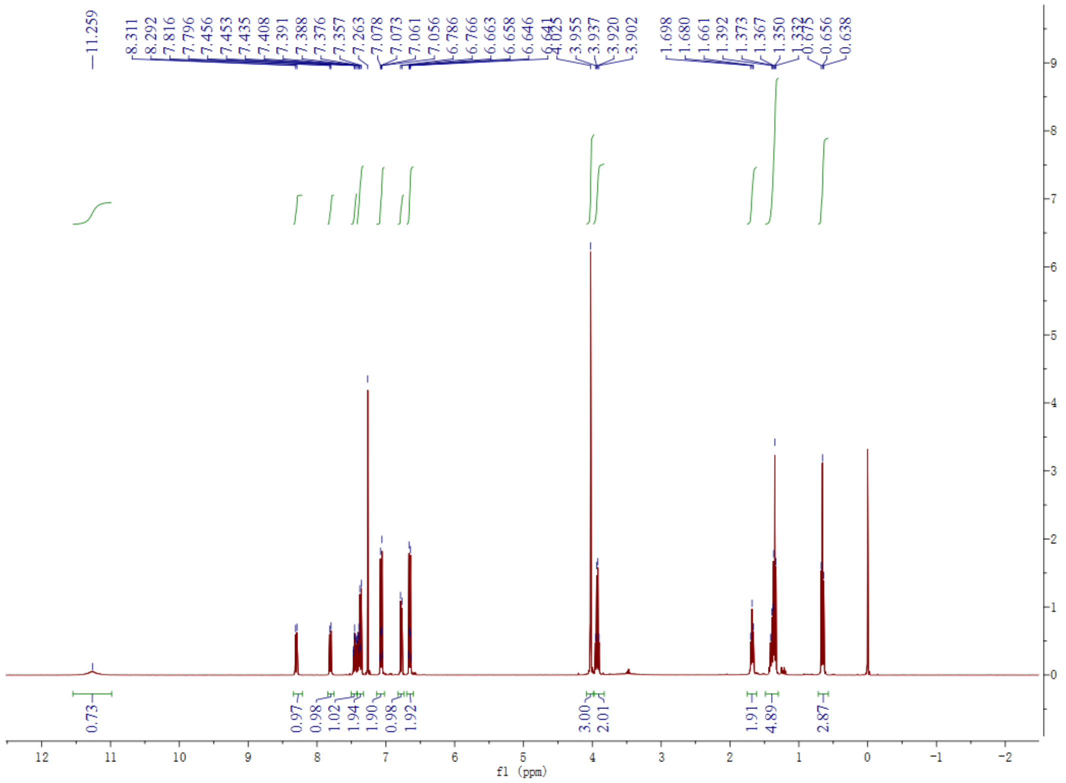

Figure S31. ^{1}H NMR of compound **6h**

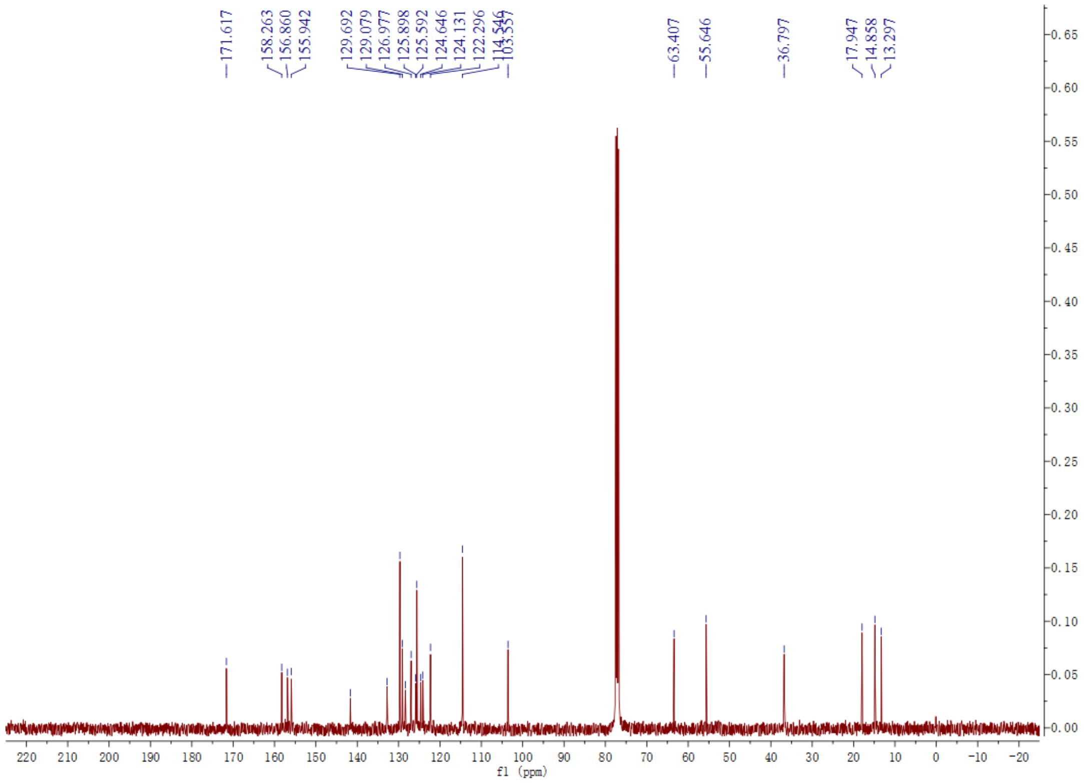

Figure S32. ^{13}C NMR of compound **6h**

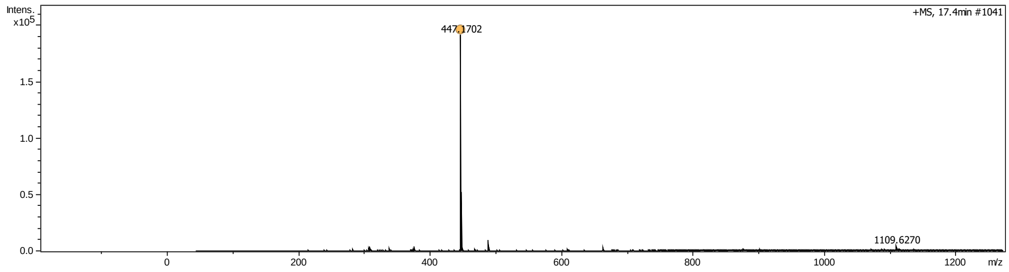

Figure S33. HRMS of compound **6h**

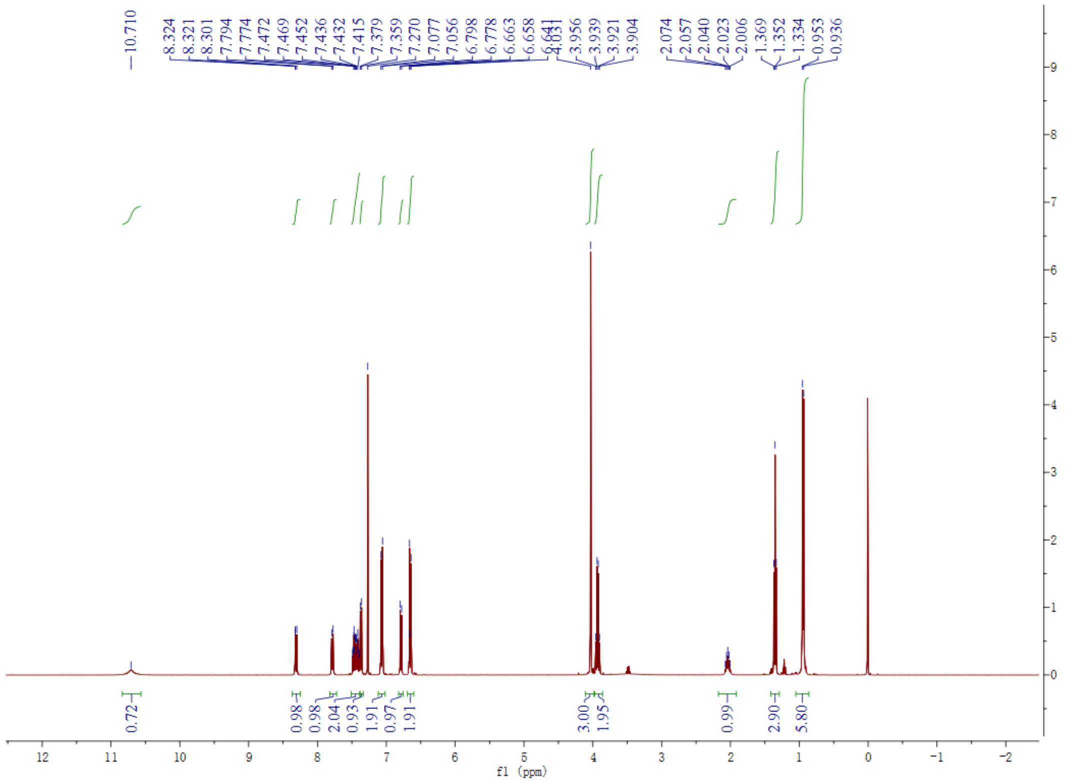

Figure S34. ^{1}H NMR of compound **6i**

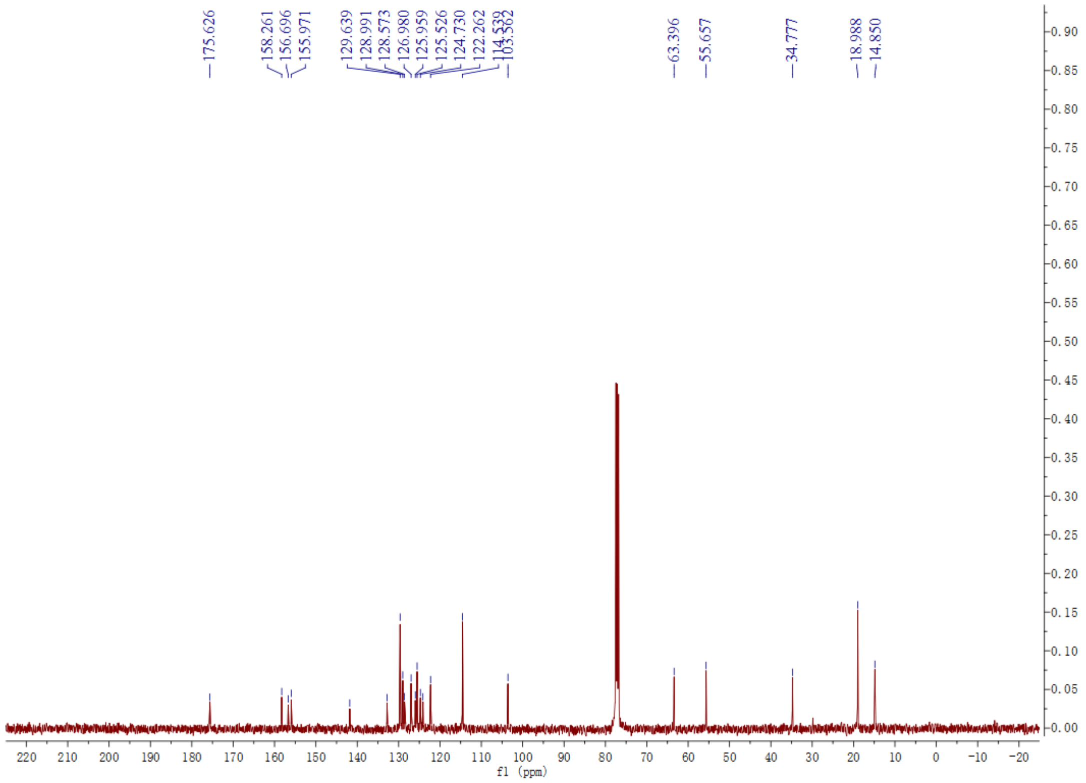

Figure S35. ^{13}C NMR of compound **6i**

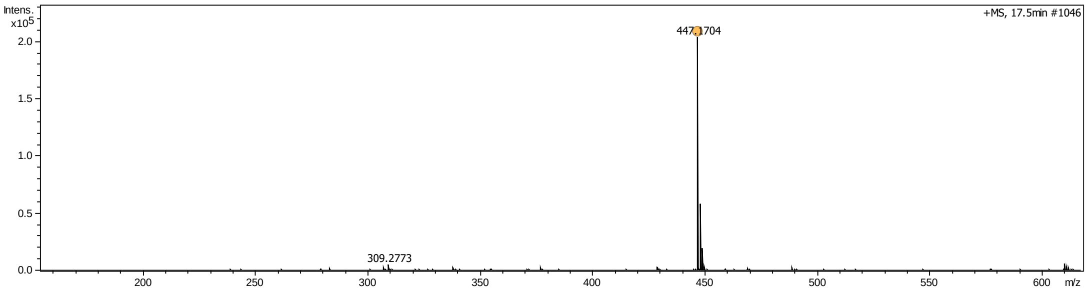

Figure S36. HRMS of compound **6i**

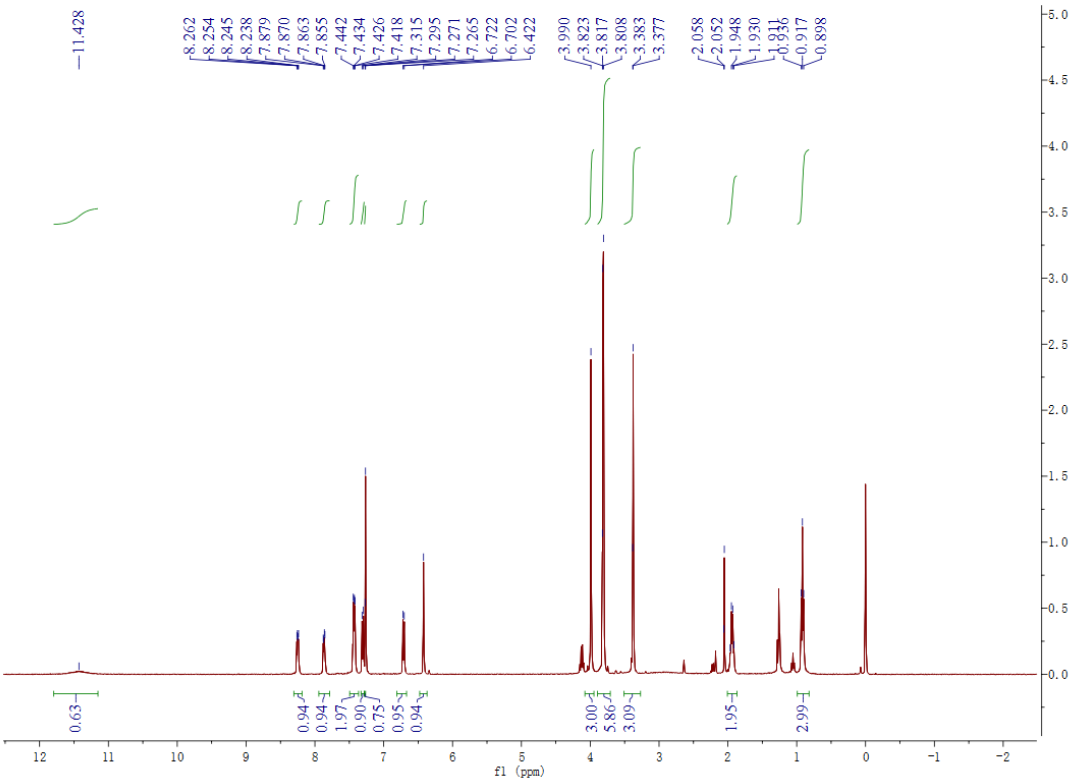

Figure S37. ^{1}H NMR of compound **6j**

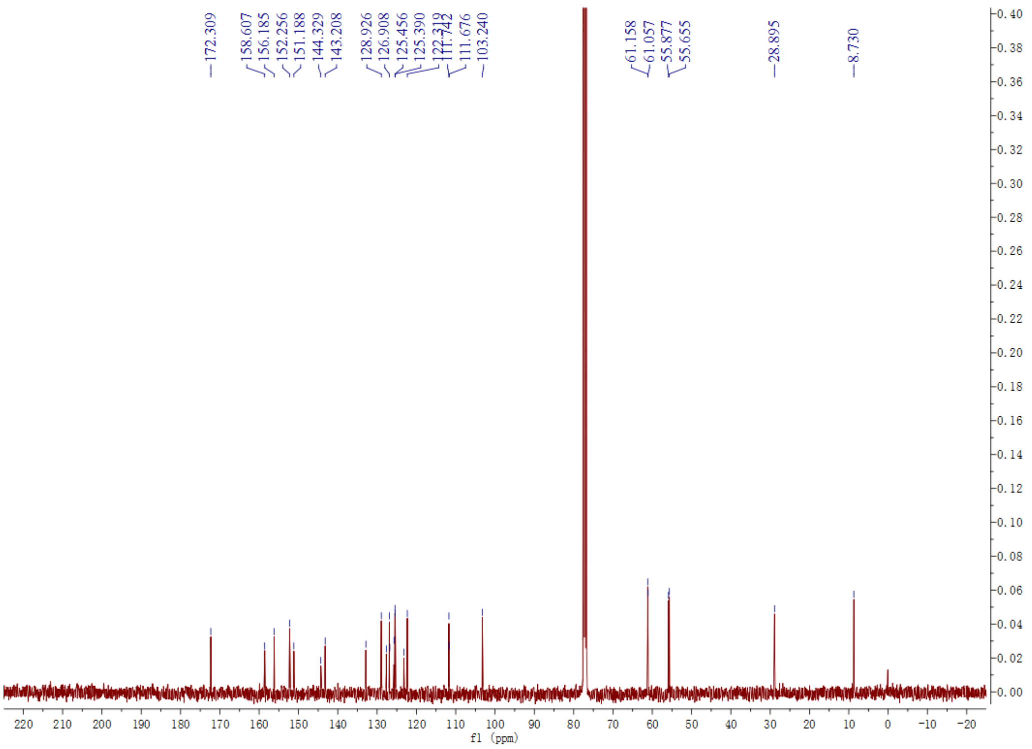

Figure S38. ^{13}C NMR of compound **6j**

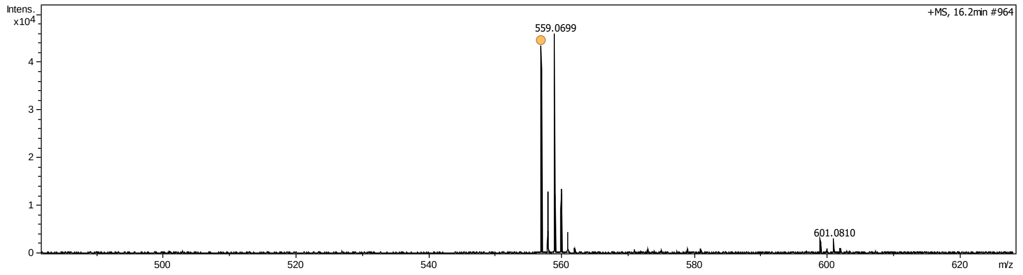

Figure S39. HRMS of compound **6j**

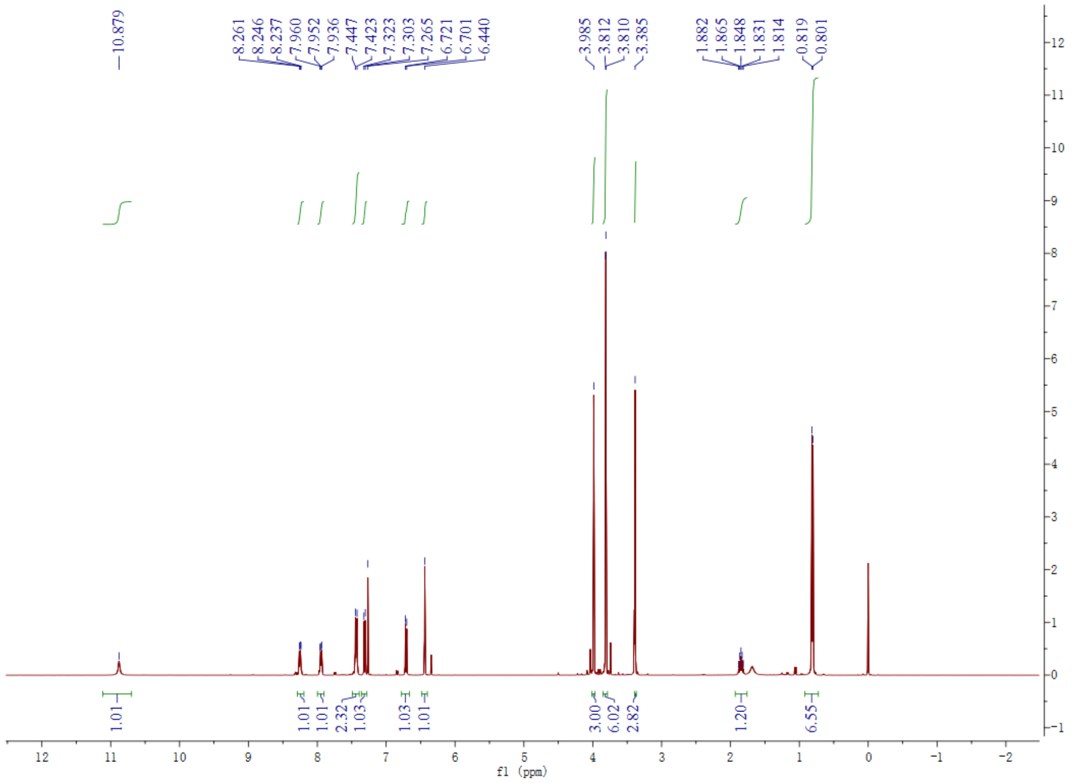

Figure S40. ^{1}H NMR of compound **6k**

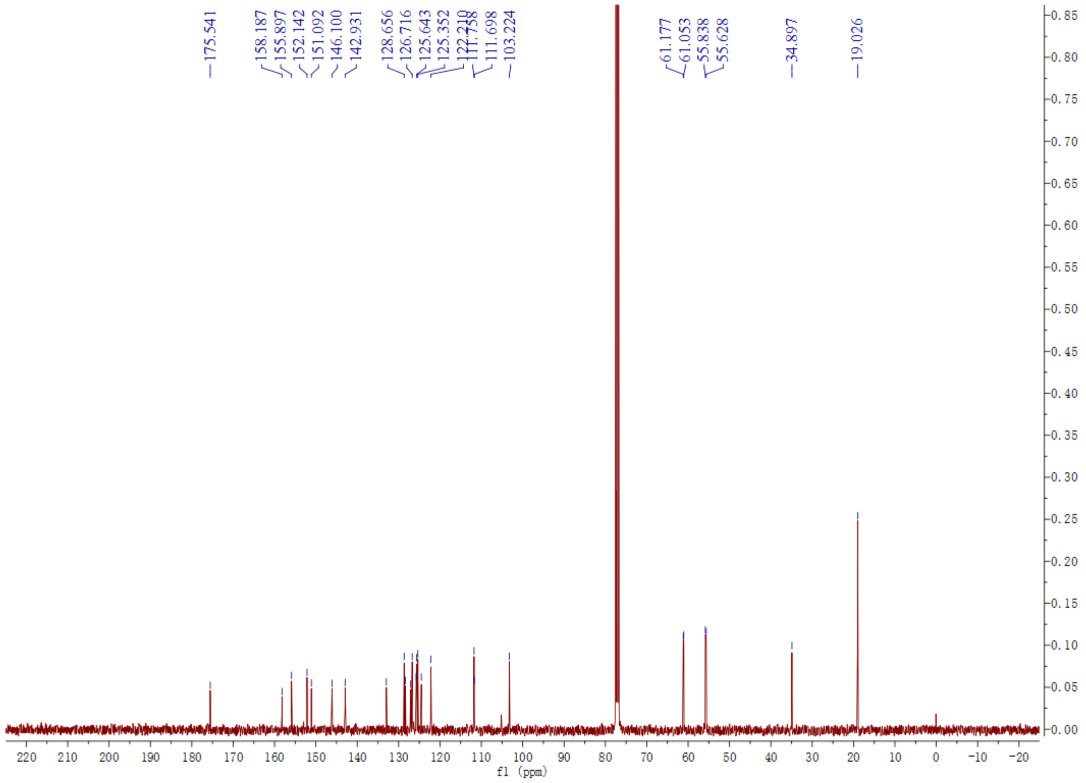

Figure S41. ^{13}C NMR of compound **6k**

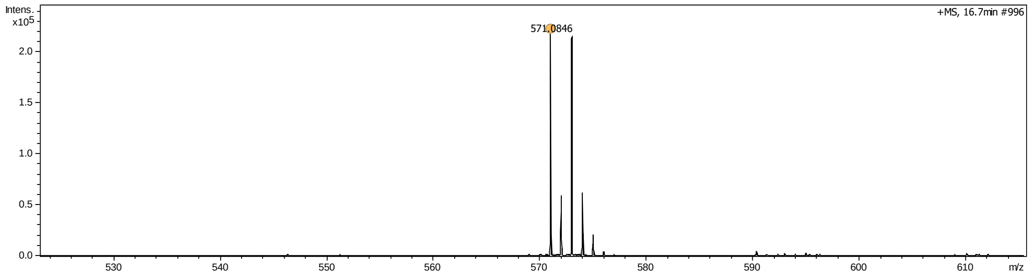

Figure S42. HRMS of compound **6k**

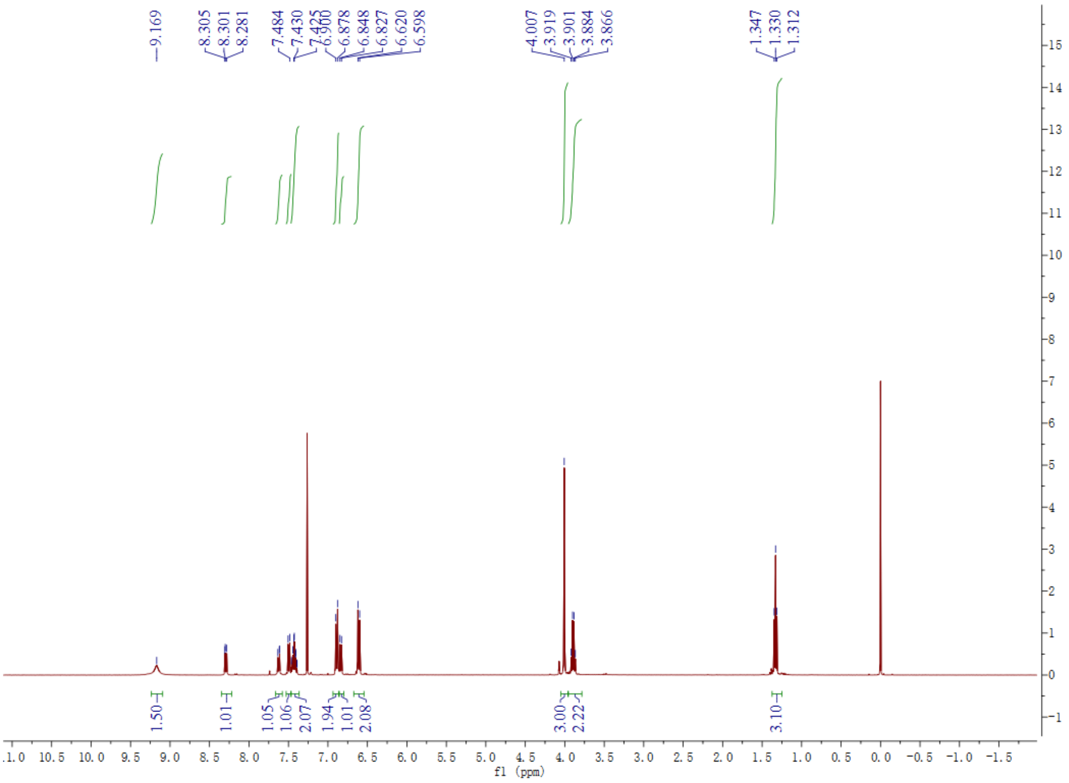

Figure S43. ^{1}H NMR of compound **6l**

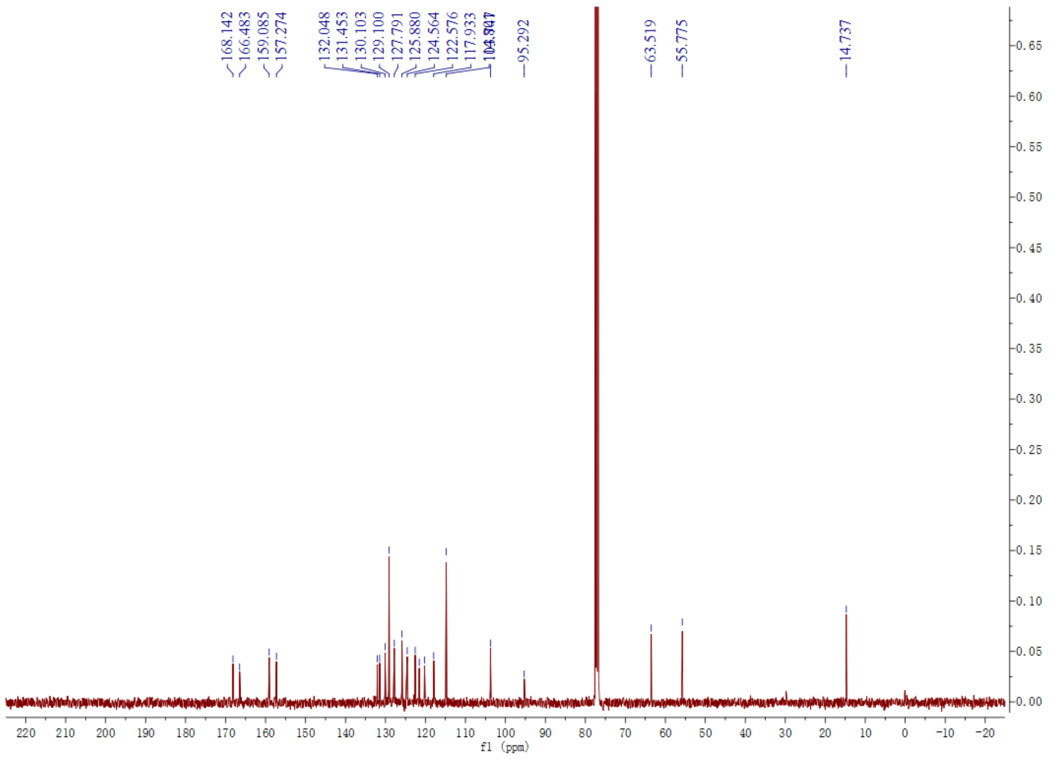

Figure S44. ^{13}C NMR of compound **6l**

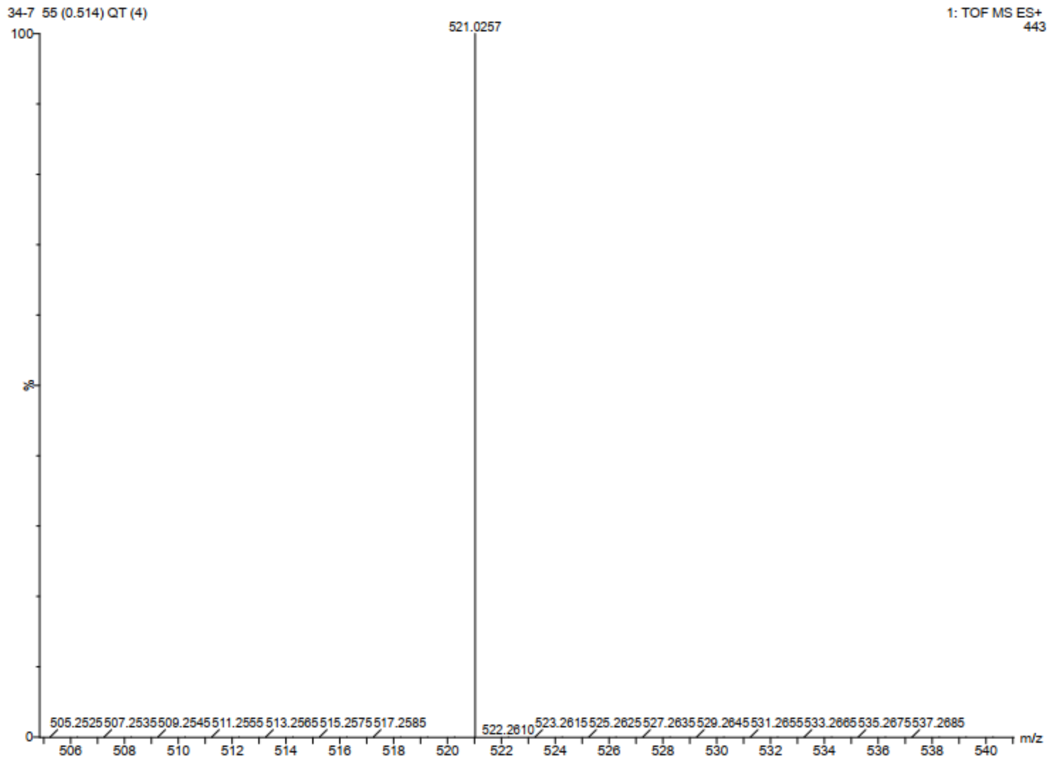

Figure S45. HRMS of compound **6l**

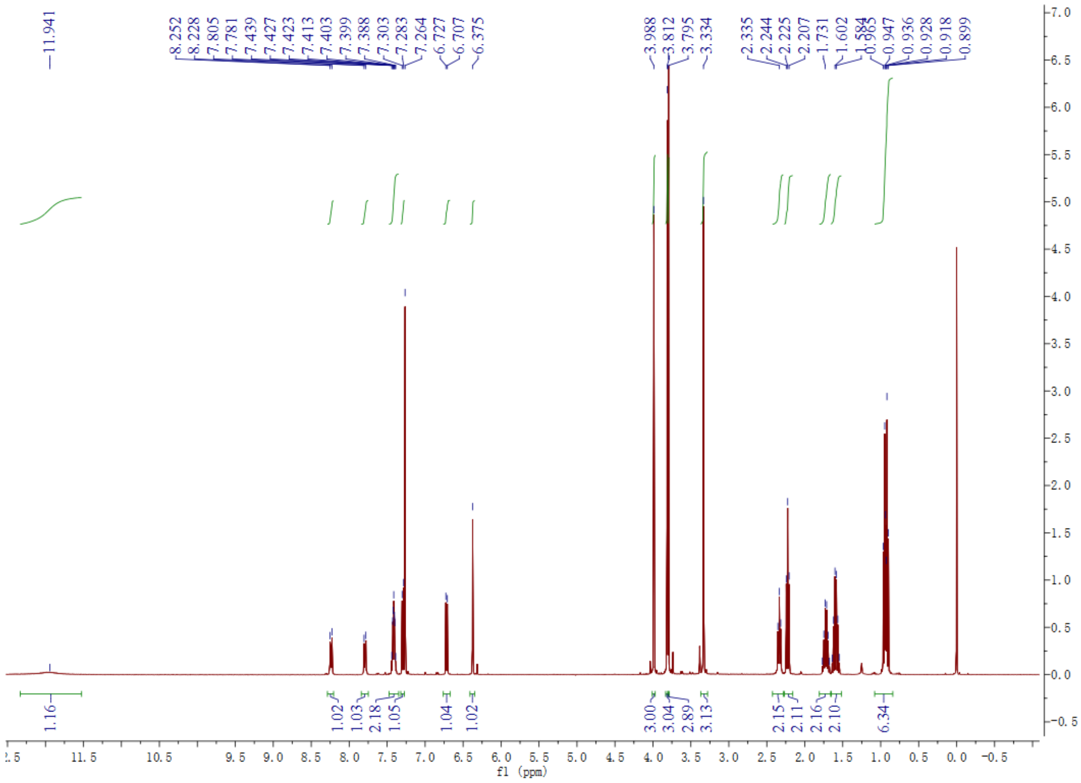

Figure S46. ^{1}H NMR of compound **6m**

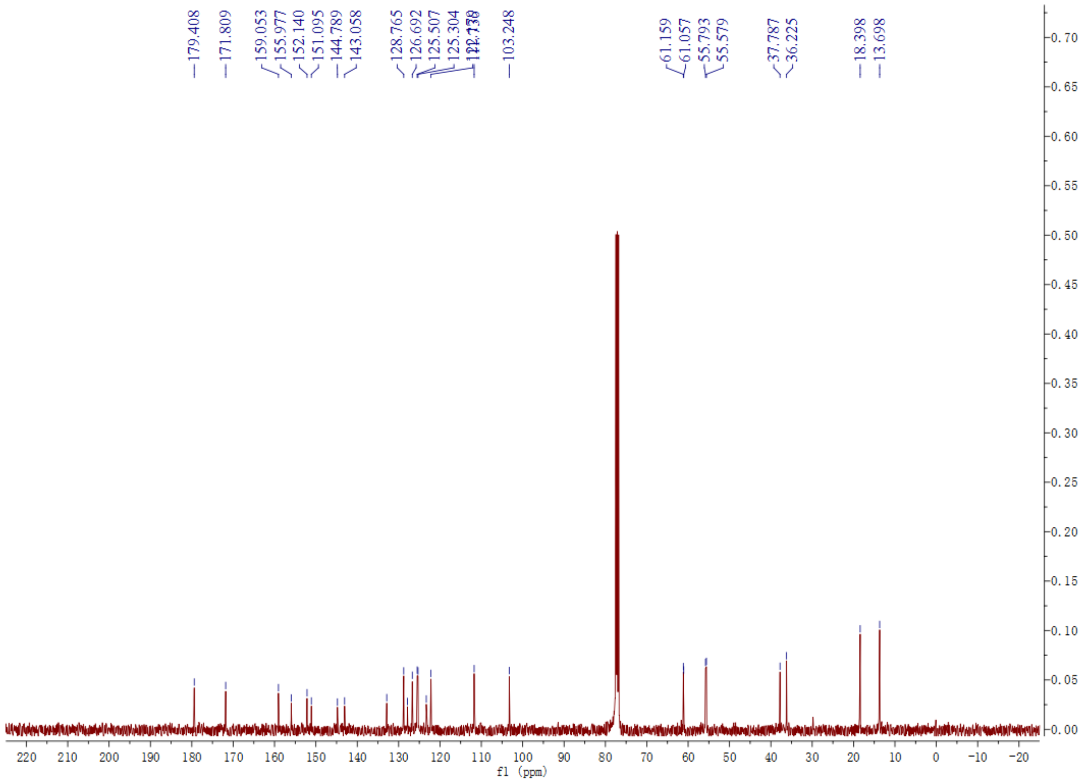

Figure S47. ^{13}C NMR of compound **6m**

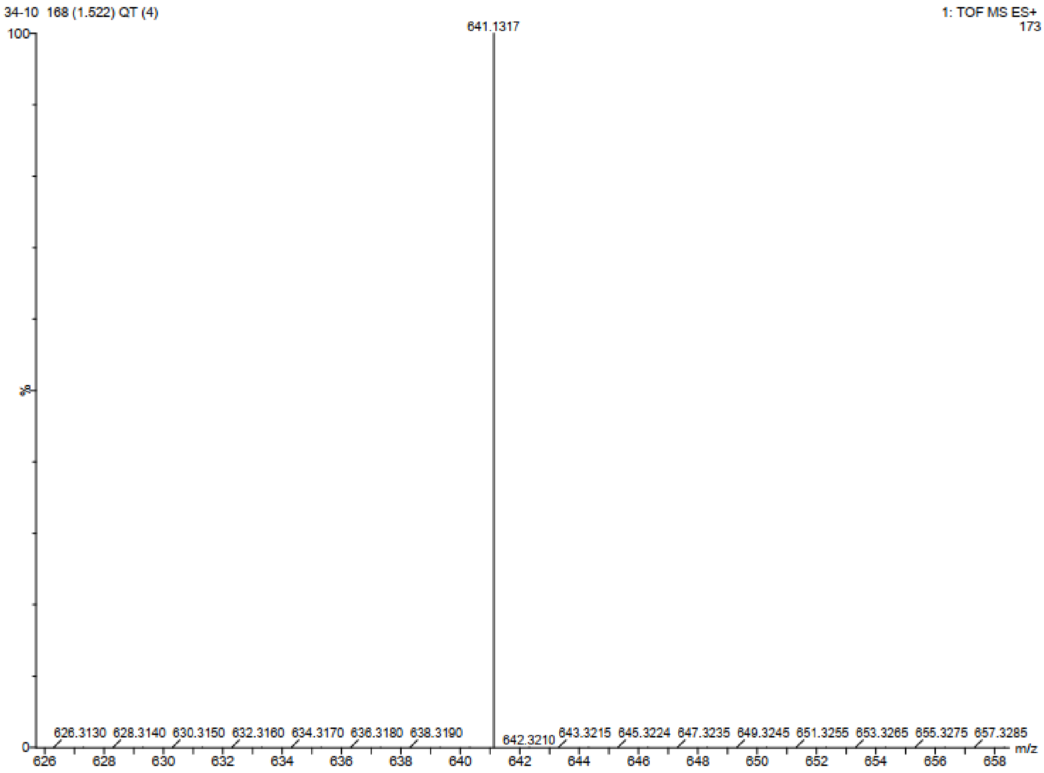

Figure S48. HRMS of compound **6m**

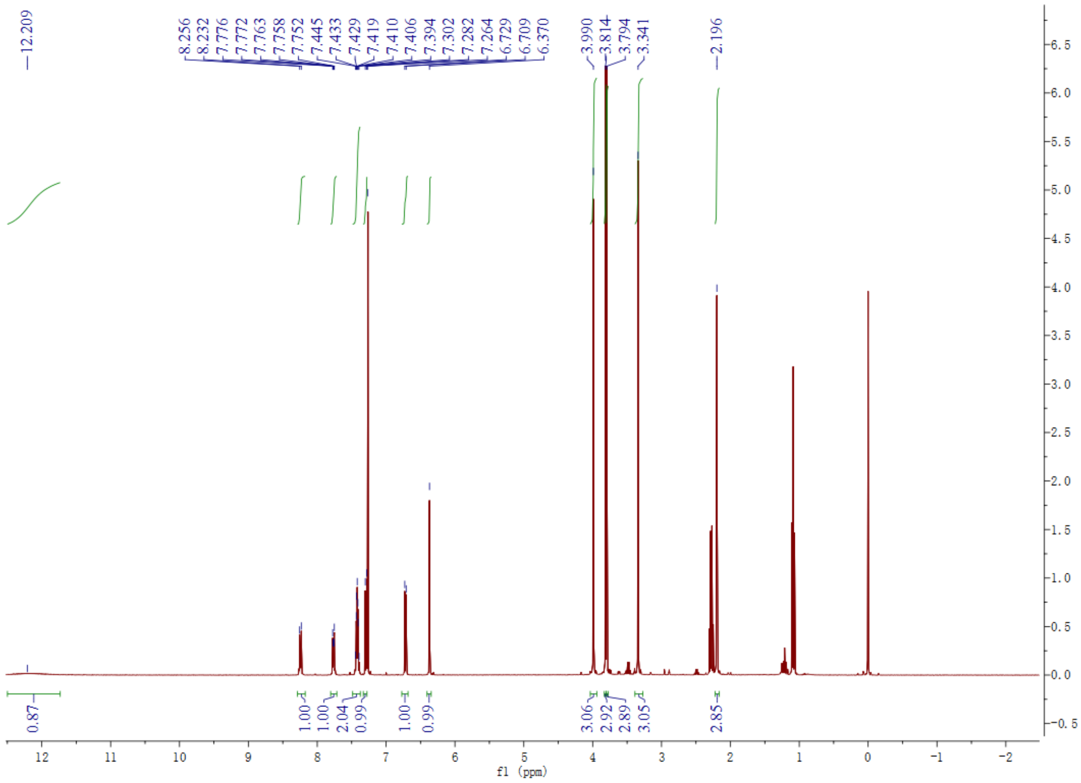

Figure S49. ^{1}H NMR of compound **6n**

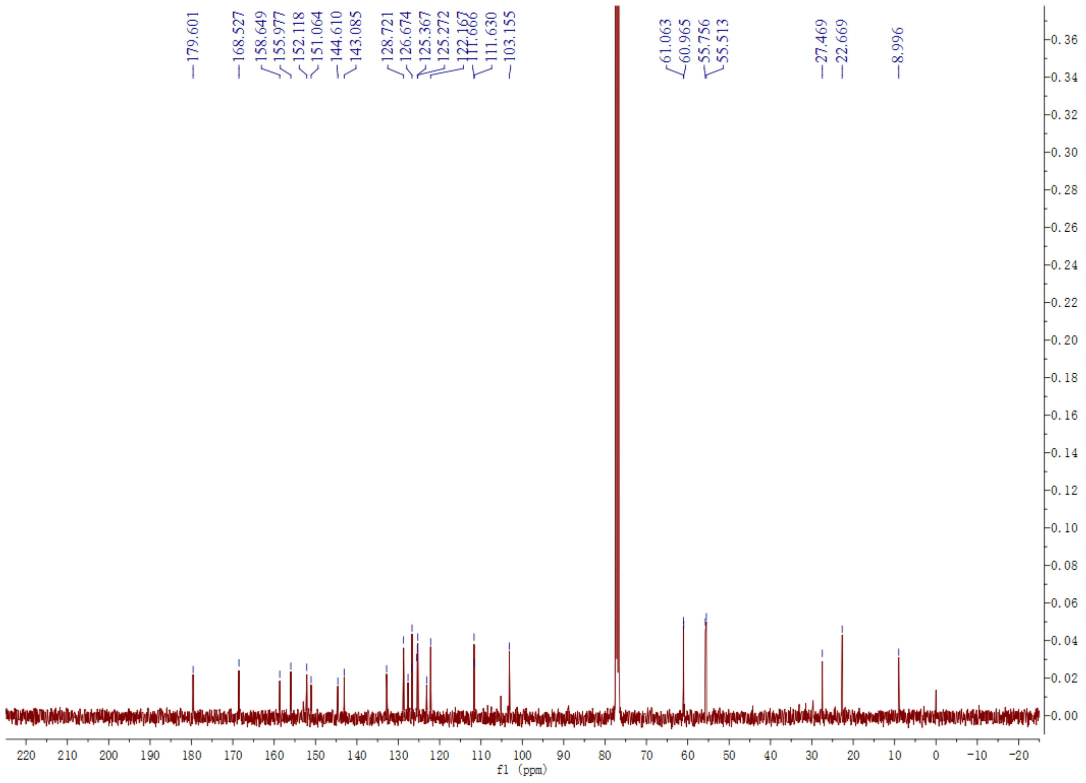

Figure S50. ^{13}C NMR of compound **6n**

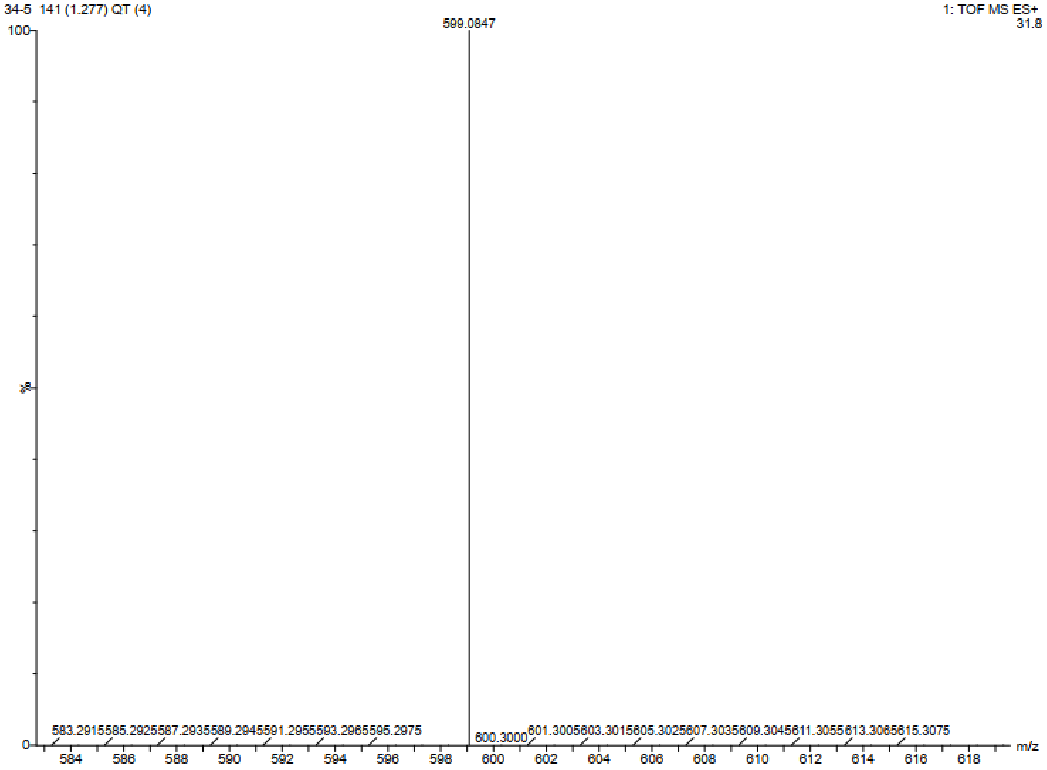

Figure S51. HRMS of compound **6n**
